# *Casuarina equisetifolia*-Derived Mesoporous Carbon for Adsorptive Removal of Phenolic Pollutants: Insights into Optimization, Isotherm, Kinetics, Thermodynamics, and Mechanism

**DOI:** 10.1021/acsomega.4c05796

**Published:** 2025-02-28

**Authors:** Praveengouda Patil, Jithu George Valiaparampil, Jaahnavi Urs, Gautham Jeppu, Chikmagalur Raju Girish

**Affiliations:** Department of Chemical Engineering, Manipal Institute of Technology, Manipal Academy of Higher Education, Manipal 576104, India

## Abstract

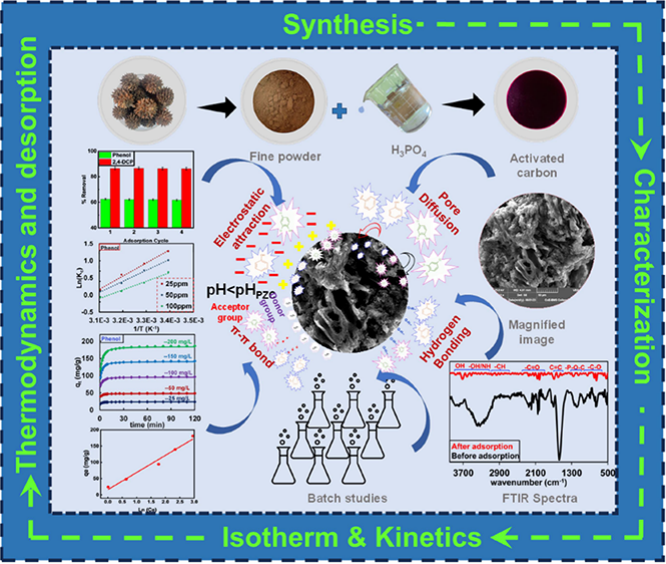

Herein, *Casuarina equisetifolia*-derived activated carbon was developed by pyrolysis at a low temperature (773 K). The derived adsorbent was characterized as an extremely porous and amorphous carbon with a surface area of 1007 m^2^/g. The point of zero charge (pH_PZC_) of activated carbon was obtained as 3.89. The reported carbon was used for the remediation of phenolic pollutants [phenol and 2,4-dichlorophenol (2,4-DCP)]. To begin with, the optimization of parameters demonstrated that a pH of 2 and a temperature of 293 K had significant impacts on the adsorption of both pollutants. The isotherm studies revealed that the Freundlich (*R*^2^ = 0.9956) and Langmuir (*R*^2^ = 0.9866) models better fitted the adsorption of phenol and 2,4-DCP, respectively. The study also reported an exceptionally elevated monolayer adsorption efficiency of 364.62 and 382.03 mg/g for phenol and 2,4-DCP, respectively. This significantly greater adsorption would aid in the elimination of phenolic pollutants, especially in coal processing industries, which are major contributors to phenolic discharge. Furthermore, kinetic studies revealed that the chemisorption mechanism dominated with *R*^2^ > 0.999 for all concentrations ranging between 25 and 200 mg/L. In addition, the thermodynamic behavior of phenol and 2,4-DCP revealed exothermic (Δ*H* < −26.70 kJ/mol) and feasible type of adsorption. The value of isosteric heat of adsorption corroborated physisorption dominating the entire process with Δ*H*_x_ < 25 kJ/mol. The adsorption mechanism aspect of phenol/2,4-DCP suggests that the removal of pollutants followed a combination of physical and chemical adsorption, accompanied by pore diffusion and electrostatic attraction coupled with π–π interaction and hydrogen bonding. The *Casuarina equisetifolia* activated carbon (CEAC) performed significantly well for four cycles of adsorption–desorption. The broader significance of these findings would yield sustainable production of *Casuarina equisetifolia*-derived carbon, which is an easily available carbon-rich source, additionally offering a cost-effective alternative solution/replacement for the remediation of phenol and chlorinated phenols from synthetic wastewater.

## 1 Introduction

Phenol, also known as carbolic acid, is an organic compound with a distinctive aroma and has significant industrial applications in pharmaceuticals^1^ and pesticides.^2^ It is a white crystalline solid at room temperature, but it also appears to be a colorless liquid. Phenolic effluent, which is a waste byproduct of industrial processes, is released into the water and poses a myriad of threats to aquatic life.^3^

The release of phenol into the water body has negative impacts on the environment, ecosystem, and public health, which stresses the value of efficient pollution control and water quality management strategies.^4^ The limit of discharge of wastewater to the waterbodies varies depending on the rules and regulations set forth by the agencies in different countries; these guidelines are imposed to protect human health, aquatic ecosystems, and the environment from the impact of pollution.^5,6^ The discharge limits by various regulating bodies are listed in Table 1.

**Table 1:** Maximum Permissible Limit of Phenol in Drinking Water and Wastewater

type/agency	World Health Organization	US Environmental Protection Agency
drinking water	0.1 mg/L	0.1 mg/L
wastewater	1 mg/L	1 mg/L

Further, the disposal of phenol in the water systems is also hazardous to the environment and ecosystem. Taking these facts into consideration and considering how toxic and harmful phenol and its derivatives are, it is necessary for these effluents to be decontaminated before releasing them into the environment. Advanced methods like oxidation processes, biological treatments, membrane processes, ion exchange, and electrochemical processes have shown promising potential in achieving the removal of phenol from wastewater.^7,8^ Adsorption is one such method that can be conducted with minimal resources and has also been implemented to remediate phenolic pollutants from wastewater. Activated carbon is a multipurpose absorbent largely used in wastewater treatment for the adsorptive removal of contaminants, including phenols and their derivatives, such as 2,4-dichlorophenol (2,4-DCP). It is known for its exceptional adsorptive capacity, is very cost-effective, and has been considered for the removal of phenols.^9,10^

Activated carbon of different types varies in its chemical and physical characteristics, which makes it suitable for specific applications and treatment scenarios. The various types that are utilized in treatments include granular activated carbon, pelletized activated carbon, powdered activated carbon, carbon block filters, and impregnated activated carbon.^11−14^

**Figure 1:**
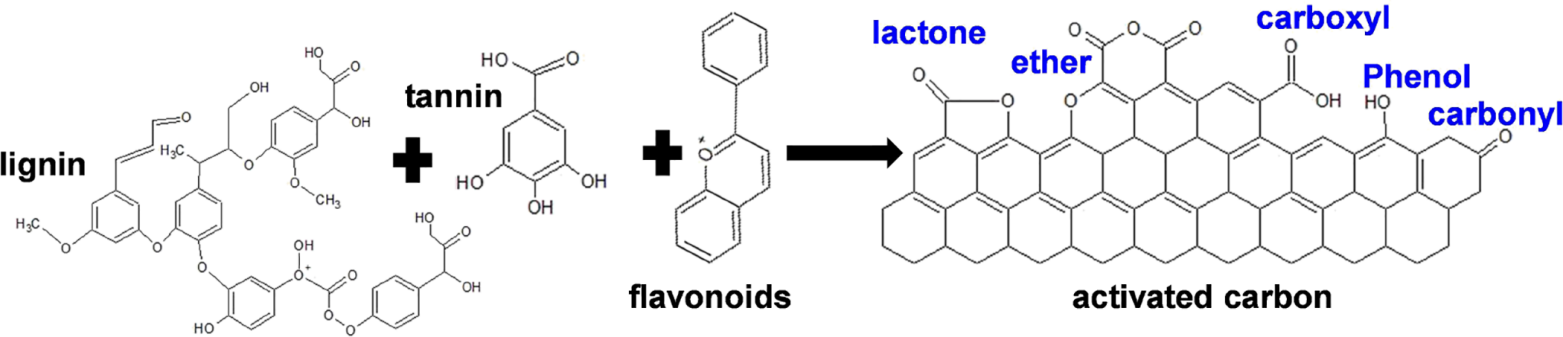
Chemical structure of the composition of *Casuarina equisetifolia* and activated carbon.

A sizable portion of the literature is available where biomass/agricultural-based activated carbon was used to eliminate phenolic wastewater.^15−17^ A few of the notable sources of agricultural waste-derived carbon are coconut shell,^11^ peanut shell,^18^ sugar cane bagasse,^19^ almond shell,^20^ bamboo,^21^ etc. *Casuarina equisetifolia* is one such source of activated carbon restricted to its availability in a few regions and has been used for the treatment of different pollutants. *Casuarina equisetifolia* shells are mainly composed of tannin, lignin, and flavonoids.^22^ This composition favors the adsorptive removal of phenolic pollutants.^9,21^ The general chemical structure of *Casuarina equisetifolia* and *Casuarina equisetifolia* activated carbon (CEAC) is shown below in Figure 1.^23^

*Casuarina equisetifolia* is a tree species that is also known as coastal she-oak and horsetail she-oak. A few more regional names are ironwood and beach she-oak and sometimes called the beach casuarina/whistling tree. The species of the tree belongs to a flowering plant in the family *Casuarinaceae*. The tree is native to countries, such as Australia, New Guinea, Southeast Asia, and India. In India, it is mostly available in the coastal regions of Karnataka, Andhra Pradesh, and Tamil Nadu.

Owing to regional availability in abundance, less usage, higher carbon content, and significant performance in abating the pollutant, the fruit, bark, needle, and leaves of *Casuarina equisetifolia* have vast relevance. From the literature, it is to be noted that for the adsorptive removal of dyes such as methylene blue, Congo red, malachite green, rhodamine B, and heavy and light oil waste, *Casuarina equisetifolia*-derived adsorbent was used.^24−28^ Apart from dyes, some authors have also employed the source for remediation of heavy metals such as copper and chromium.^29−31^ However, to the best of our knowledge, studies related to the one-pot synthesis of *Casuarina equisetifolia* seeds are limited, and the adsorption of phenol/2,4-DCP by *Casuarina equisetifolia* seeds is unexplored.

So, this research paper adopts an academically aggressive stance and emphasizes decontaminating synthetic wastewater. It further aims to encourage sustainable water management methods and highlights the flexibility of activated carbon in phenol/2,4-DCP removal. The objectives of the investigation are (i) to synthesize *Casuarina equisetifolia*-derived activated carbon, (ii) to optimize adsorption operational parameters for the removal of phenolic pollutants, and (iii) to study isotherms, kinetics, thermodynamics (isosteric heat of adsorption), and mechanism.

## 2 Methodology

### 2.1 Preparation and Measurement of Pollutant

The chemicals phenol, 2,4-DCP, sodium hydroxide, and hydrochloric acid were procured from Merck, India. A stock solution of 500 mg/L was prepared by adding and mixing a certain amount of crystalline phenol to 1000 mL of distilled water taken in a standard flask of 1000 mL capacity. Further, the stock was diluted to different concentrations ranging from 25 to 200 mg/L and was used for future studies. The concentration of phenol and 2,4-DCP was measured using a UV–vis spectrophotometer (UV-1900i, Shimadzu) at 270 and 283 nm, respectively.^32,33^

### 2.2 Synthesis of *Casuarina equisetifolia*-Derived Activated Carbon

The procedure to synthesize activated carbon was referred from our previous studies with some modifications.^34^ The dry seeds of *Casuarina equisetifolia* were collected from the MIT, Manipal campus, Karnataka. Initially, 200 g of seeds was thoroughly washed with deionized (DI) water. Further, the washed samples were placed in an oven for a period of 24 h at 378 K for drying. The moisture-free samples were crushed into a fine powder and sieved through a 425 μm sieve. Then, 15 g of the fine powder was mixed manually with 15 mL of orthophosphoric acid and allowed to stand for 6 h. The impregnated/mixed sample was equally transferred to 25 mL capacity silica crucibles and kept for the carbonization process at 773 K for 60 min in the presence of N_2_ gas. Further, the activated sample was cooled and washed with 1–2% sodium bicarbonate solution until a neutral pH was achieved. The sample with nearly a neutral pH was then dried in a hot air oven at 100 °C for 24 h. This sample was later transferred to an airtight container and labeled as *Casuarina equisetifolia* activated carbon (CEAC). The above procedure was repeated two to three times, and the samples were checked for surface area (SA) and pore volume (PV) to check the reproducibility of the activated carbon. CEAC was further characterized and used for other investigations.

### 2.3 Characterization of Activated Carbon

The prepared CEAC was characterized by using several sophisticated techniques such as BET, FTIR, XRD, and SEM-EDS. A surface analyzer (Smart Sorb, Smart Instruments) was used to identify the SA and PV at −196 °C. The X-ray diffractometer was used (Rigaku Miniflex 600, fifth gen) with a step size 5°/min (2θ ranging between 10 and 60°) to identify the crystalline/amorphous structure of CEAC. The porosity and elemental details of the adsorbent were measured by SEM-EDS (EVO MA18 with Oxford EDS, X-act) at 750× magnification. Further, the functional groups that could contribute to the adsorption of pollutants were studied using the FTIR technique (ATR-FTIR, Shimadzu-8400S) in the range 400–4000 cm^–1^.^35−37^ In addition, the thermal stability of the CEAC for temperatures ranging from 298 to 973 K was also investigated for the sample weighing 9.58 mg and heated at 10 °C/min by maintaining an inert atmosphere by using thermogravimetric analysis (TA 55 discovery, TA Instruments Austria).

Furthermore, to identify the sort of the dominant charge of the functional groups present on CEAC, point of zero charge (pH_PZC_) experiment was performed for an initial adjusted pH ranging from 2 to 12.^34^ The study would be helpful in the adsorption of charged pollutants. Here, a certain amount of adsorbent was continuously stirred in a temperature-controlled shaker for 24 h. The final pH after 24 h was measured and plotted as final pH versus initial pH.

### 2.4 Batch Tests-Optimization of Operating Factors

Before proceeding to isotherm, kinetic, and thermodynamic studies, one needs to understand the crucial operating parameters that govern the whole adsorption process. Therefore, each parameter (contact time, dosage, pH, temperature, and concentration) is optimized.^39^ Initially, the adsorption tests were run to identify the possible contact time required for the entire adsorption process. Here, 50 mL of the diluted pollutant of concentration 50 mg/L was transferred to a 250 mL conical flask with 50 mg of CEAC and kept in a thermostat-shaker with a shaking speed of 120 rpm and a temperature of 30 °C for 6 h. Further, in another set of studies, the dosage was varied from 10 to 100 mg. The pH of the working solution is tuned (2–12) using dilute acid and base.^10^ The temperature was manually changed, ranging between 293 and 323 K. The range of the concentrations considered in the current investigation was from 25 to 200 mg/L. After equilibrium was attained, 2 mL of sample from each conical flask was collected in airtight clean vials and allowed to centrifuge for 10 min. The centrifuged samples were used to calculate the residual concentration and the removal efficiency as given by eq 1. Further, the experimental adsorption capacity (*q*_exp_) was quantified using eq 2
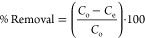
1
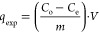
2In the above equations, *C*_o_ and *C*_e_ are the initial and equilibrium concentrations of the pollutant in mg/L, “*m*” is the CEAC dose in mg, and “*V*” is the volume of the pollutant in mL considered during the experiment.

### 2.5 Isotherm, Kinetics, and Thermodynamics Studies

Soon after identifying the optimized parameters, different isotherm models such as Langmuir, Freundlich, Redlich Peterson (RP), Temkin, and Dubinin–Radushkevich (DR) models were investigated to figure out the possible type of adsorption leading to the adsorption of both the pollutants. While Langmuir, Freundlich, and RP isotherms can be applied to confirm monolayer or multilayer adsorption,^33^ Temkin and DR models were used to compute the adsorption energy parameters. The linear forms of the isotherms were applied to quantify the adsorption ability (*q*_e_) and other parameters eqs 3–8^6,40,41^
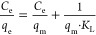
3
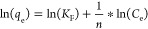
4
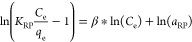
5*q*_m_ is the theoretical maximum adsorption ability at equilibrium (mg/g), *K*_L_ is related to the Langmuir adsorption constant in L/mg, *K*_F_ is the Freundlich model constant in L/g, and “*n*” is dimensionless constant.^42^ Further, *K*_RP_ and *a*_RP_ are constants related to the RP model.^33,43^
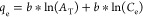
6
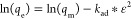
7
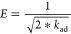
8In the above equations, *b* represents the Temkin constant which relates to the heat of adsorption (J/mol) and *A*_T_ signifies the equilibrium binding constant (L/g). In addition, *k*_ad_ refers to the activity coefficient related to the adsorption energy (mol^2^/kJ^2^), ε is the Polanyi potential (kJ/mol), and “*E*” from the DR model is the average free energy (kJ/mol).^44^

The best model that fits the experimental values was chosen based on the highest *R*^2^ value. In the case of kinetic experiments, the investigation was performed for varied concentrations ranging from 25 to 200 mg/L until equilibrium was accomplished. The study involves drawing 2 mL of aliquots every 5 min until equilibrium contact time. The experimental values were further fitted to different available kinetic models.^39^ Pseudo-first-order (PFO) and pseudo-second-order (PSO) are few of the most applied models, especially to quantify the rate and the type of adsorption mechanism (physisorption/chemisorption) that is dominating the adsorption.^45^ The equations, i.e., eqs 9 and 10 mathematically represent PFO and PSO as given. Further, the degree of mean absolute % error was also evaluated by using eq 11.
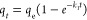
9
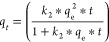
10
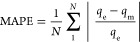
11In the above equations, *q_t_* and *q*_e_ are the adsorption capacity at time “*t*” and the theoretical adsorption capacity in (mg/g). *q*_m_ is the model adsorption capacity (mg/g). Further, *k*_1_ in 1/min and *k*_2_ in g/mg/min denote the rate constants from PFO and PSO equations.^34^

In thermodynamics, batch tests were accomplished at different temperatures ranging from 293 to 323 K. The investigations were carried out basically to understand if the process of adsorption was exothermic/endothermic by means of enthalpy.^46^ The calculations related to enthalpy change (Δ*H*^o^), entropy change (Δ*S*^o^), and Gibb’s free energy (Δ*G*^o^) are also quantified using standard equations as mentioned below, which could further be used to compute the degree of spontaneity and feasibility of adsorption.^47^ In addition, the isosteric heat of adsorption was also computed to identify the dominating adsorption process in the removal of the pollutant^48^ by making use of the Clausius–Clapeyron equation. This would also serve as a confirmation of the thermodynamics study. The Clausius–Clapeyron equation is given below in eq 14
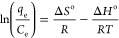
12
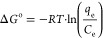
13
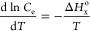
14In the above eqs 12–14, Δ*S*^o^ represents the entropy change during the adsorption process (kJ/mol/K), Δ*H*^o^ is the enthalpy change (kJ/mol), Δ*G*^o^ is the Gibbs free energy (kJ/mol), *R* is denoted as the universal gas constant (J/mol/K), and *T* is the temperature (K).

### 2.6 Desorption and Reusability Study

The reusability of CEAC was tested by performing desorption experiments. An adsorption study is followed by desorption of phenol/2,4-DCP from the surface of the material. In the case of phenol, a total of six flasks, each filled with 25 mg/L, dosage of 2 g/L, was set up at the optimum conditions as mentioned in previous sections and allowed to run for 180 min. A similar set of experiments was also executed for the adsorption of 2,4-DCP at 50 mg/L and a dosage of 0.4 g/L. Further, the unadsorbed pollutant was analyzed in a UV–vis spectrophotometer. Then, the CEAC particles were separated from the aqueous solution and allowed to dry in a hot air oven at 378 K. Further, the dried carbon particles were transferred to three to four flasks containing 0.1 N NaOH solution. The setup was agitated for 1 h at room temperature to facilitate desorption/separation of the pollutant and activated carbon. Then, the desorbed solution was decanted, and 50 mL of distilled water was added to each flask and agitated for 2 h. The distilled water would assist in removing additional pollutants from the surface of CEAC and neutralize the surface. Further, the CEAC was separated and dried in a hot air oven. The procedure was repeated for four to five cycles. The plot of removal efficiency versus number of cycles is available in subsequent sections. The desorption efficacy can be computed using the equation eq 15
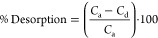
15In the above equation, *C*_a_ is the amount of adsorbate present in the solid phase and defined as the difference between the initial and equilibrium concentrations (mg/L) and *C*_d_ is the amount of adsorbate present in the liquid phase/eluent (mg/L).

## 3 Results and Discussion

### 3.1 Mechanism of Pore Formation and Characterization of CEAC

#### 3.1.1 Mechanism of Synthesis of Porous CEAC

Orthophosphoric acid (H_3_PO_4_) is considered one of the most used activating agents. H_3_PO_4_ supports the pyrolytic conversion of the source and promotes reorganizing the cross-linked structure within the carbon-rich material.

As per the literature,^49,50^ three different mechanisms are involved when H_3_PO_4_ interacts with the biomass carbon source. In the first stage, the decomposition of higher molar mass organic matter to lower molar mass material is accompanied by the discharge of gaseous and volatile substances. The second stage is characterized by the beginning of the breakdown of H_3_PO_4_ along with the distribution of intermediate compounds, which also involves tar and a fraction of coal formation. In the final stage, the creation/formation of pores is a result of char interaction with P_2_O_5_.

In the literature, the pyrolysis temperature range of 673–973 K, in addition to phosphoric acid, is commonly preferred.^37,51^ However, some studies have also reported the application of higher pyrolysis temperatures >973 K for the preparation of activated carbon.^52,53^ Based on the temperature of pyrolysis, step-by-step reactions occur between 273 and 673 K, and some occur between 673 and 973 K. The reactions are mathematically represented below from eqs 16–20. They demonstrate the changes that occur in the acid (H_3_PO_4_) during the pyrolysis process until 973 K only as we are working at a pyrolysis temperature of 773 K. In the first stage, i.e., between 373 and 673 K, the acid undergoes dehydration, accompanied by a breakdown function because of the release of carbon dioxide and carbon monoxide.
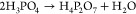
16
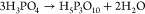
17
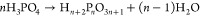
18

In the second stage, i.e., between 673 and 973 K, a strong oxidant is formed that reacts with carbon, resulting in the formation/widening of pores, the creation of new functional groups, and the release of unwanted gases.
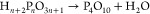
19
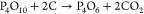
20

#### 3.1.2 BET, XRD, and Analysis of Functional Groups

**Figure 2:**
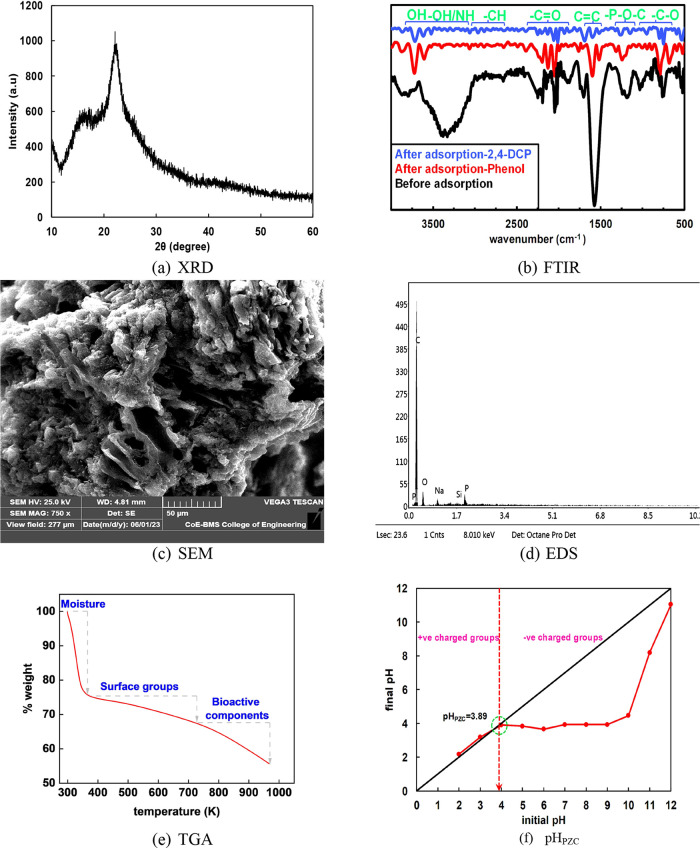
Structural and morphological characterization of CEAC. (a) XRD. (b) FTIR. (c) SEM. (d) EDS. (e) TGA. (f) Point of zero charge of CEAC.

From the BET analyzer, a higher surface area of 1007 m^2^/g and a pore volume of 0.6521 m^3^/g was obtained. The material can be termed as mesoporous as the pore diameter is greater than 2 nm and less than 50 nm. The peak of Figure 2a shows distinct and broad peaks at 15°, 23°, and 45°. These peaks correspond to planes of graphite at 002, 100, and 110. The intense peak of the XRD spectrum at 23° depicts the presence of an amorphous structure in CEAC. Similar peaks and patterns are also reported for novel carbon prepared from *Ceiba speciosa*.^54^ The dominance of amorphous nature in the CEAC would favor higher removal of phenolic pollutants in the present investigation.^12^

FTIR studies of CEAC were performed for pre- and post-adsorption (Figure 2b). The significant functional peaks that would contribute to adsorption were determined using FTIR. It can be observed that there is a broad peak between 3700 and 3000 cm^–1^, which indicates the existence of hydroxyl (−OH/NH) functional groups at 3380 cm^–1^ in activated carbon.^55,56^ Further, the peaks corresponding to −CH vibration were found between 3000 and 2500 cm^–1^, indicating the presence of aliphatic compounds.^57^ In addition, the peak at 2250 cm^–1^ is an indication of the occurrence of carbonyl group (C=O).^18,58^ Furthermore, a greater peak at 1610 and 1210 cm^–1^ displays the presence of aromatic compounds and alkoxy groups, respectively.^17^ In addition, the minor peaks between 1200 and 980 cm^–1^ at 1210 cm^–1^ depict phosphorus-containing carbon.^59^ Similar findings were also documented for rice husk-activated carbon treated with orthophosphoric acid.^14,60^ The alkane peak bending vibrations (C=C) may be seen at a peak around 964 cm^–1^.^61^ Post adsorption of phenol/2,4-DCP spectra, peaks were diminished at 3750, 2250, 1800, 1590, and 1250 cm^–1^ revealing active participation of the functional groups by facilitating different mechanisms such as hydrogen bonding and π–π interaction. The detailed adsorption mechanism is discussed in the following section.

#### 3.1.3 Morphology, Composition, and Thermal Stability of CEAC

The structural and morphological characteristics of CEAC were obtained from SEM analysis. Figure 2c demonstrates a magnified image of carbon particles. The impact of acid impregnation and thermal treatment can be undoubtedly observed as there is formation of tubular/elongated channels.^47^ These densely spaced varied pore developments confirm that the structure could be more porous with higher SA and improved adsorption efficacy of the adsorbent.^62^ Similar observations were reported for porous activated carbon derived from *Toon sinensis* leaves.^63^

The presence of different possible elements detected in CEAC was determined via EDS investigation, as shown in Figure 2d. A higher intensity of carbon (81.78%) can be observed which demonstrates that the precursor “*Casuarina equisetifolia*” considered for the investigation was rich in carbon molecules. Further, smaller peaks of phosphorus (0.84%) were due to acid impregnation carried out during the synthesis of carbon, which was negligible. In addition, the trace amount of sodium (1.05%) was due to the usage of dilute sodium bicarbonate for washing and silica (0.15%) depicting the presence in the raw material (details provided in Table 2).

**Table 2:** EDS Analysis of CEAC for Elemental Composition

elemental composition of CEAC		
element	weight %	atomic %
C	81.78	86.21
O	16.18	12.81
Na	1.05	0.58
Si	0.15	0.07
P	0.84	0.34

The graph of %weight versus temperature is illustrated in Figure 2e. The figure indicates nearly 24.8% weight loss until 378 K which was mainly due to moisture loss.^64^ This moisture loss is an indication of the presence of physically bound water molecules on the adsorbent.^65^ In the second stage, between 378 and 773 K, approximately 9.391% of the mass was reduced, and the loss was due to the degradation of hemicellulose, lignin, and cellulose.^54^ Additionally, losses witnessed until 773 K could be attributed to the loss of bioactive components. In the end, 44.283% of CEAC was retained depicting the presence of carbon in the precursor.

#### 3.1.4 Analysis of Point of Zero Charge

The point of zero charge (pH_PZC_) of CEAC is a balance point where the number of positive and negative sites of carbon are nearly equal.^33^Figure 2f shows the graphical representation of pH_PZC_ for CEAC. It can be noted that the pH_PZC_ of the carbon was found to be approximately 3.89. Further, it depicts that the carbon surface will be majorly surrounded by positive charge when the pH of the solution is less than pH_PZC_ and vice versa. pH_PZC_ will play a substantial role in the removal of phenolic pollutants.

### 3.2 Results of Optimization of Operational Parameters

#### 3.2.1 Impact of Contact Time and Dosage

**Figure 3:**
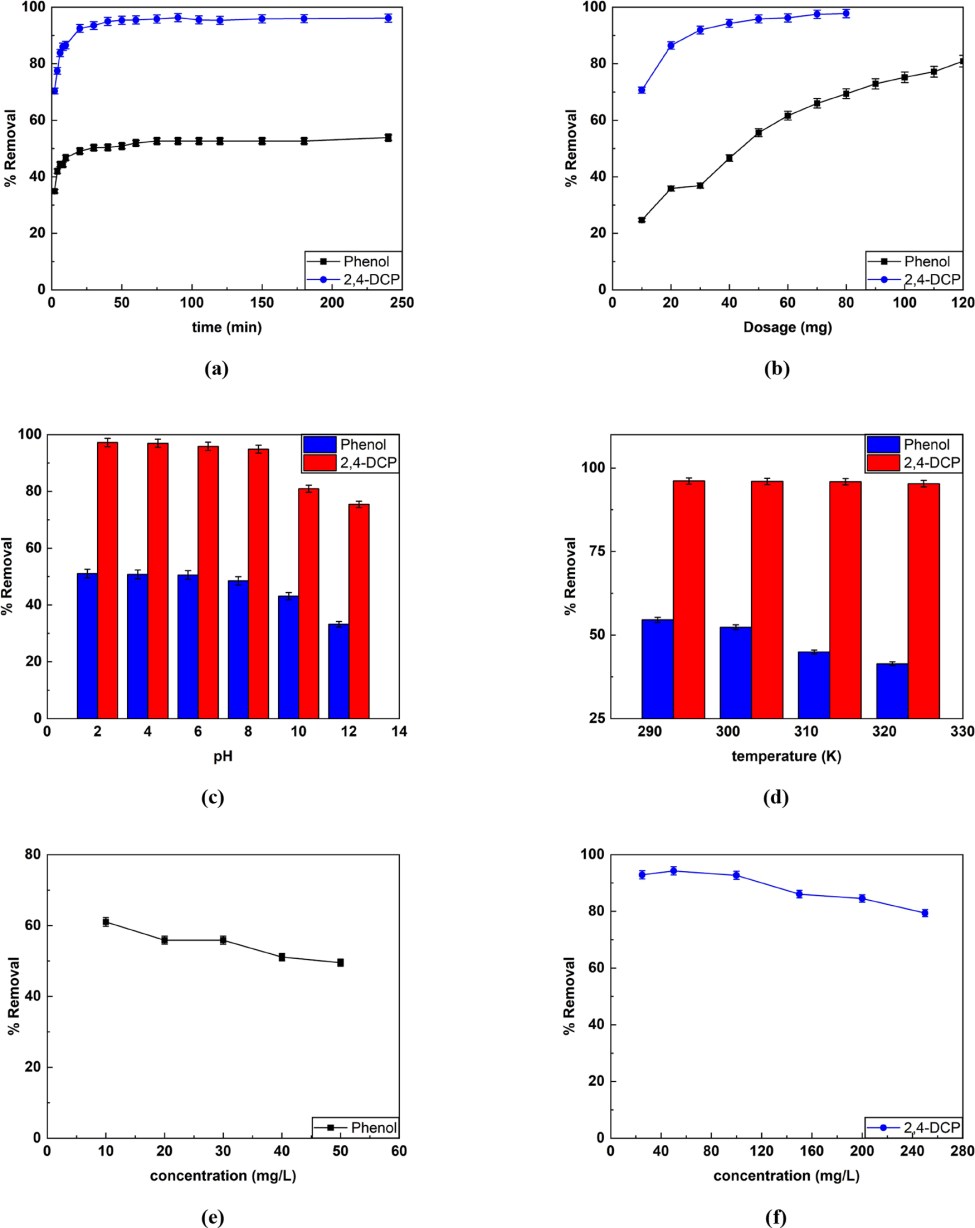
Impact of operational parameters on the adsorption of phenol and 2,4-DCP. (a) Contact time, (b) dosage, (c) pH, (d) temperature, (e) concentration of phenol, and (f) concentration of 2,4-DCP.

Adsorption studies were investigated for the removal of phenols onto CEAC for a duration of 240 min. The experimental setup involved was dosage 1 g/L, speed 150 rpm, pH 2, and temperature 303 K. The plot of equilibrium time versus removal efficiency is shown in Figure 3a.

In the case of phenol adsorption, it could be observed that the removal efficiency increased from 0 to 45% in the first 10 min. Further, the remaining phenol was adsorbed until equilibrium was attained in 240 min. However, removal in the case of 2,4-DCP reached 86% within 10 min. The remaining 10% of the 2,4-DCP concentration was able to adsorb onto CEAC in 240 min. Thus, for both studies, 240 min was considered the equilibrium contact time.

Figure 3b illustrates the impact of sorbent dosage on the removal of pollutants. In the case of phenol, the dosage was varied from 0.2 to 2 g/L. The experimental setup involved speed 150 rpm, pH 2 and temperature 303 K, and *t* 240 min. However, in the case of 2,4-DCP, the dosage was varied from 0.2 to 1.6 g/L.

In both cases, it can be noted that the removal efficacy increased with an increase in the CEAC dosage. This rise was due to the increased number of active sites available for adsorption.^66,67^ In addition, the sites were unadsorbed after an increase in the additional dose of the adsorbent. Therefore, in the case of phenol, 2 g/L was considered as the optimum dosage as the % removal was near 75%, and a further increase in dose would not yield significant adsorption. Meanwhile, the optimized dosage finalized for 2,4-DCP removal was 0.8 g/L due to the highest percentage removal of 94% and significant adsorption ability.

#### 3.2.2 Impact of pH of Solution and Temperature

pH and temperature play vital roles in terms of higher adsorption of pollutants. The batch tests were conducted for the adsorption of phenol and 2,4-DCP for varying pH (2–12). The impact on the removal of contaminants is illustrated in Figure 3c. The other experimental conditions are dosage 1 g/L, speed 150 rpm, temperature 293 K, and *t* 240 min.

In both cases, it can be visualized that the removal efficacy in acidic conditions, specifically at pH 2, was higher. The pH at which the adsorbent surface possesses zero charge is known as the point of zero charge (pH_PZC_). pH_PZC_ of CEAC occurs at 3.89, Figure 3f. When pH is decreased to <3.89, positive charges are formed, and pH > 3.89, it facilitates negative charge on the CEAC surface. In other words, phenolic compounds are mostly available in protonated form, backing more adsorption. So, at lower/acidic pH, phenol exists in pure molecular form. These conditions support the development of electrostatic forces of attraction between CEAC and phenols.^68^ Further, phenol being a weak acid with p*K*_a_ = 9.89 gets dissociated when the pH of the solution exceeds p*K*_a_. With the surge in pH values, the removal efficiency of phenol subsides. This descent is due to the ionization of phenol molecules and repugnance action between anionic phenolate and negatively charged CEAC sites.^69,70^ Similar phenomena are also observed in the case of the adsorption of 2,4-DCP.

Figure 3d demonstrates the plot of %removal versus temperature for adsorption of phenol and 2,4-DCP. The experimental settings considered are dosage 1 g/L, speed 150 rpm, temperature 293–323 K, pH 2, and *t* 240 min. It can be observed that higher phenolic compound removal was observed at lower temperatures, and removal efficiency was depleted with an increase in temperature. This could be due to a decline in attractive forces/bond strength between CEAC and phenols.^40^ Similar trends have been reported by other researchers.^71^ Meanwhile, no significant effect was observed in the adsorption of 2,4-DCP.

#### 3.2.3 Impact of Initial Concentration

The effect of varying concentrations on CEAC adsorption was studied. The plot of the same is shown in Figure 3e,f. The tests for phenol adsorption were conducted for concentrations ranging from 10 to 50 mg/L and for 2,4-DCP which was varied between 10 and 250 mg/L. The other experimental parameters are dosage 1 g/L, speed 150 rpm, temperature 303 K, pH 2, and *t* 240 min. In both investigations, an inverse relation of removal efficiency to increased concentration was observed. The reason for the fall was due to the absence of active CEAC sites for the adsorption of pollutants.^72^

### 3.3 Isotherm, Kinetics, and Thermodynamics Studies

#### 3.3.1 Equilibrium Modeling for Phenol and 2,4-DCP

**Figure 4:**
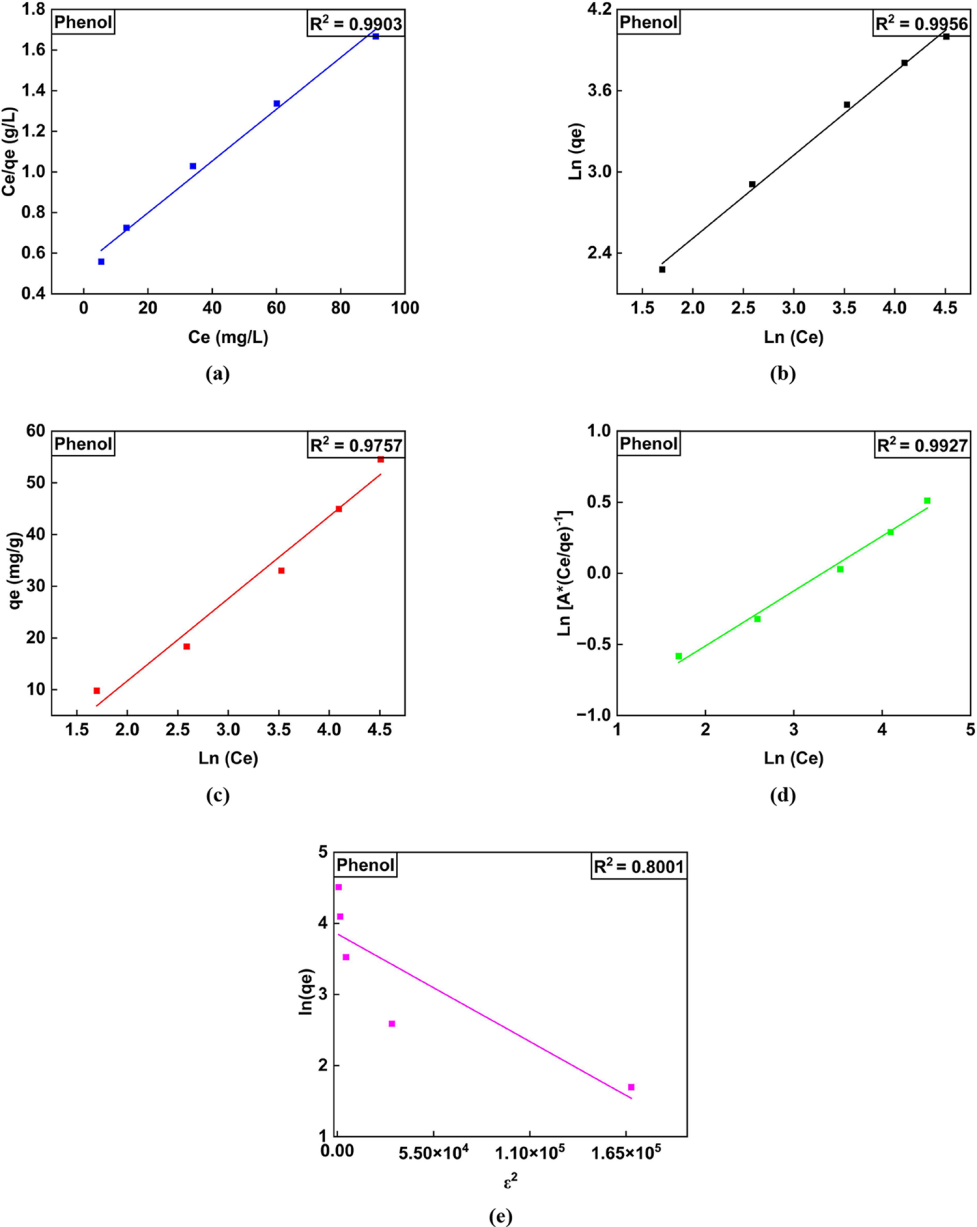
Linear isotherm plots for the adsorption of phenol. (a) Langmuir, (b) Freundlich, (c) Temkin isotherm, (d) RP model, and (e) DR model for quantification of isotherm parameters.

**Figure 5:**
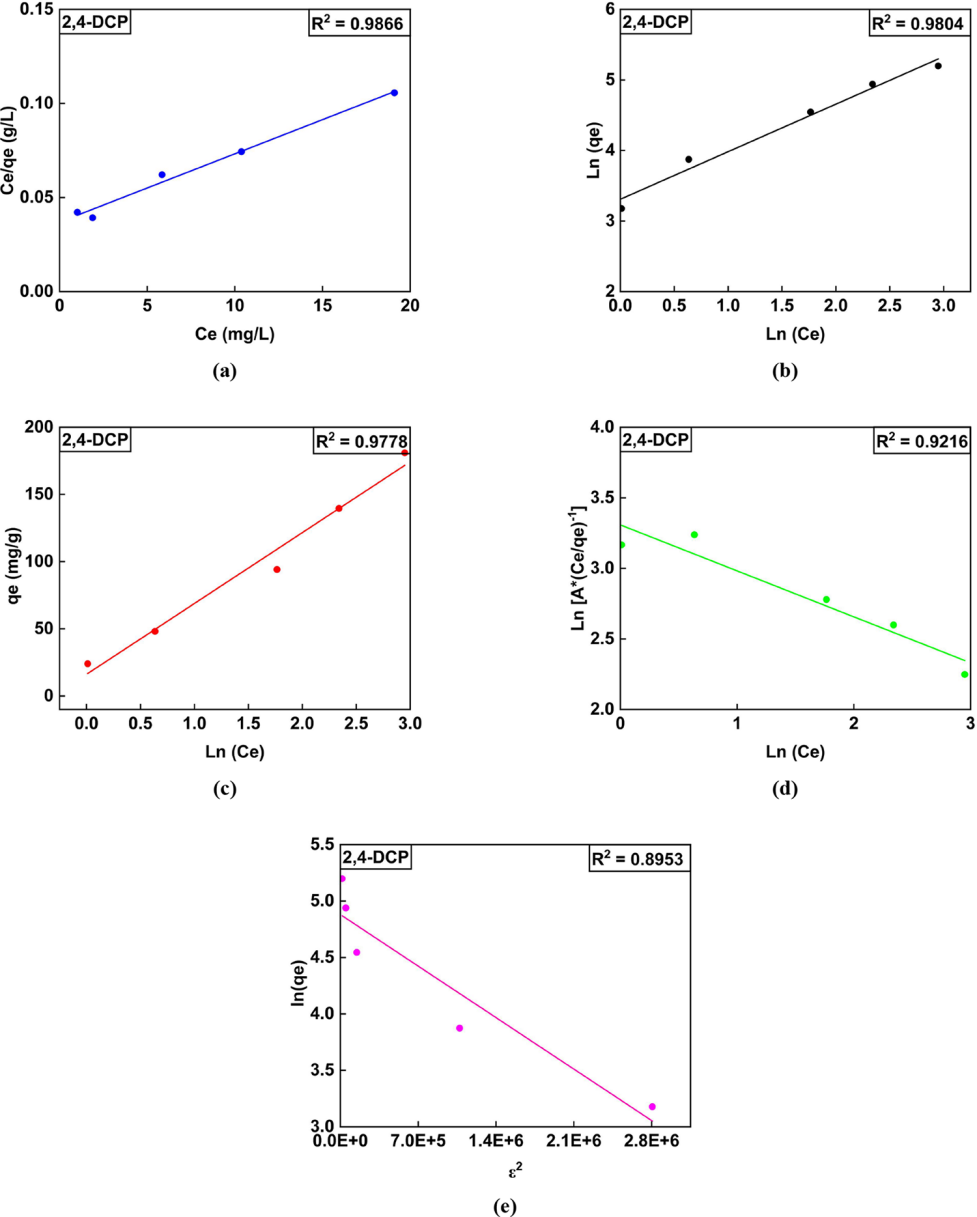
Linear isotherm plots for the adsorption for 2,4-DCP. (a) Langmuir, (b) Freundlich, (c) Temkin isotherm, (d) RP model, and (e) DR model for quantification of isotherm parameters.

The linear isotherm modeling for phenol and 2,4-DCP was investigated for concertation ranging from 25 to 200 mg/L. The graphical representation of all isotherms is illustrated in Figures 4 and 5.

A linear graph of *C*_e_/*q*_e_ versus *q*_e_ was plotted. The obtained linear equation was used to determine the Langmuir model parameters for the adsorption of pollutants. The monolayer adsorption capacity for phenol and 2,4-DCP was found to be 364.62 and 382.03 mg/g, respectively. The additional isotherm parameters are listed in Table 3. The Langmuir constant “*K*_L_” can be used to quantify the favorable shape factor/feasibility parameter “*R*_L_,” the range of which varies from 0 to 1.

**Table 3:** Isotherm Parameters for the Adsorption of Phenol and 2,4-DCP on CEAC

isotherm models	parameters	units	phenol	2,4-DCP
Langmuir model	*q*_m_	mg/g	364.62	382.03
	*K*_L_	L/mg	0.003	0.0909
	*R*^2^		0.9903	0.9866^a^
Freundlich model	*n*	constant	1.1566	1.6046
	*K*_F_	(mg/g) (L/mg)^1/*n*^	1.6077	41.013
	*R*^2^		0.9956^b^	0.9804
Temkin model	*A*_T_	L/g	0.1932	1.3587
	*B*	J/mol	21.500	77.004
	*R*^2^		0.9757	0.9778
RP model	*K*_RP_	L/g	4.4489	29.05
	*a*_RP_	(L/mg) ^β^	1.9171	0.0299
	β	constant	0.1681	1.311
	*R*^2^		0.9927	0.9216
DR model	*k*_ad_	mol^2^/K^2^ J^2^	9 × 10^–06^	7 × 10^–07^
	*E*	kJ/mol	235.70	845.15
	*R*^2^		0.8001	0.8953

a Langmuir model better fitted 2,4-DCP adsorption.

b Freundlich model better fitted phenol adsorption.

The sorption feasibility is possible if 1 > *R*_L_ > 0 and non-favorable and irreversible if *R*_L_ > 1 or *R*_L_ = 0. Herein, the *R*_L_ varied from 0.93 to 0.62 (for phenol) and 0.305 to 0.05 (for 2,4-DCP).^6,36^ The range of values in the case of phenol and 2,4-DCP adsorption thus depicts the process as desirable and reversible.

Further, a linearized equation from the plot of ln (*q*_e_) versus ln (*C*_e_) was employed to compute the Freundlich adsorption constant of both pollutants. It could be confirmed that the process is desirable/feasible at ambient temperature as the value of 1/*n* varies between 0 and 1.

Temkin parameters were obtained from plot *q*_e_ against ln (*C*_e_). The parameter *A*_T_ is related to the maximum binding energy. Higher *A*_T_ values indicate stronger interactions between the adsorbate and the adsorbent. In this case, *A*_T, 2,4-DCP_ > *A*_T, phenol_ implies that 2,4-DCP (1.3587 L/g) has a stronger interaction with the CEAC than phenol (0.1932 L/g). Further, parameter “*b*” depicts the change in adsorption energy as a function of surface coverage. Higher values of “*b*” indicate a substantial decline in the heat of adsorption with increasing coverage, suggesting more considerable adsorbate–adsorbent interactions. For 2,4-DCP, the greater value of *b* = 77.004 implies that the heat of adsorption decreases more drastically with increasing surface coverage compared to phenol.

In addition, the parameter (*A*_RP_) in the RP model depicts the adsorption affinity of CEAC toward phenol and 2,4-DCP. The *A*_RP_ value of 2,4-DCP was 6.52-fold higher than that of phenols. Thus, this demonstrates a greater affinity of the adsorbent towards 2,4-DCP. The exponent “β” for phenol was found to be approaching “0,” and in the case of 2,4-DCP, the value is close to 1. The β value of phenol and 2,4-DCP suggests the possible occurrence of heterogeneous and homogeneous adsorptions, respectively.^73^

Furthermore, from the DR model, higher values of sorption energy for phenol, i.e., *E*_phenol_ = 235.70 kJ/mol, and for 2,4-DCP i.e., *E*_2,4-DCP_ = 845.15 kJ/mol, were observed. These values were greater than 8 kJ/mol suggesting that the chemisorption type of mechanism was prevalent.^9^ In addition, Freundlich^72^ and Langmuir model^61^ best described the adsorption process of phenol and 2,4-DCP, respectively. Based on the *R*^2^ value, the order of fit was Freundlich > RP model > Langmuir > Temkin isotherm > DR model in the case of phenol. Further, for 2,4-DCP, the order was Langmuir > Freundlich > Temkin > RP model > DR model.

Data from all of the different biomass-derived sources and their respective adsorption capacities are shown in Table 4. It can be observed that CEAC has categorically performed well in most of the cases by exhibiting a comparatively higher adsorption capacity for both phenol and 2,4-DCP. Based on these data, it is also evident that some materials demonstrated a higher adsorption ability than CEAC. However, CEAC stands out as one of the options for treating phenolic pollutants owing to the regional availability and ease of production of carbon.

**Table 4:** Comparison of Experimental Conditions and Adsorption Capacity with Other Research Works

		experimental conditions							
pollutant	adsorbent source	contact time (min)	operation speed (rpm)	concentration (mg/L)	adsorbent dose (g/L)	pH_solution_	best fit isotherm model	adsorption capacity (mg/g)	ref.
phenol	activated coke	180	200	25–100	1	2	Langmuir	14.39	(15)
	olive branch	120		2.5–7.5	2	8	Langmuir	58.82	(16)
	banyan root	60	100	20–100	2.5	7	Langmuir	26.95	(40)
	olive stone	720		250–500			Freundlich	120	(74)
	Brazil nuts shell	180	150	25–200	0.75	6	Sips	99	(75)
	*Acacia mangium*		150	50–150	2	3	Langmuir	53.8	(76)
	oak wood	90	200	10–150	1		Freundlich	108.7	(77)
	date kernel	100	200	5–50	5	4	Langmuir	5.988	(78)
	*Tithonia diversifolia*	60	180	100–500	6	8	Langmuir	50.55	(66)
	rice husk	120	150	1–5	2	6	Langmuir	103.9	(79)
	*Cassia fistula* pods	180	120	25–500	1.6	2	Redlich Peterson	183.79	(34)
	*Casuarina equisetifolia*	180	150	25–200	2	2	Freundlich	364.62	this work
2,4-dichlorophenol	*Populus nigra*	2800	160	20–500	2	6	Freundlich	105	(33)
	bamboo sheath	40	180	30–120	7	2	Langmuir	37.29	(61)
	pine saw dust	400	120	25–100	0.75	4	Freundlich	135.7	(70)
	cocoa pod husks	180	120	10–50	1	7	Langmuir	37.45	(46)
	black tea leaves	180	75	80–800	10	2	Langmuir	45.5	(80)
	wood-based activated carbon	90	80	100–500	0.25	7.27	Langmuir	204.08	(81)
	bamboo charcoal	100	120	0.5–100	5	2	Freundlich	45.25	(82)
	*Oryza sativa*	10	100	10–100	0.04	1	Langmuir	156.48	(83)
	*Cassia fistula* pods	120	150	25–500	0.6	2	Langmuir	374.4	(34)
	*Casuarina equisetifolia*	180	150	25–200	0.8	2	Langmuir	382.03	this work

#### 3.3.2 Analysis of Desorption and Regeneration of CEAC

**Figure 6:**
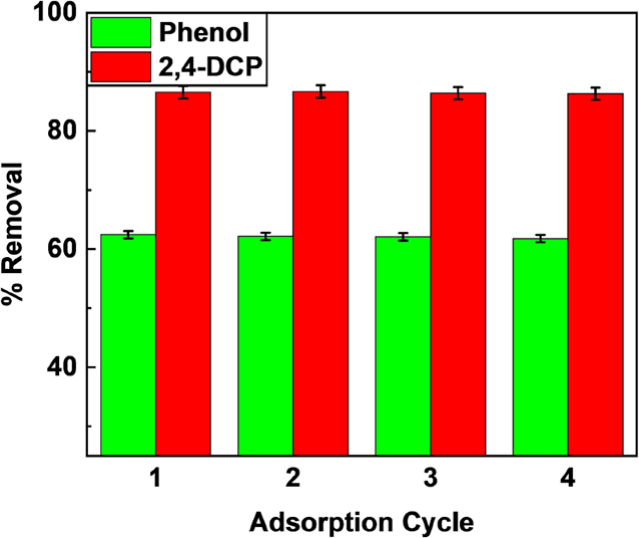
Recyclability of CEAC (Experimental conditions: Phenol/2,4-DCP, pH= 2.0, dosage = 2 g/L/0.8 g/L, *T* = 293 K, *C*_o_ = 25 mg/L/50 mg/L).

To investigate the reusability and stability of CEAC, 0.1 N NaOH was chosen as the eluent/desorbing agent. From Figure 6, it can be observed that the removal efficiency was nearly constant. For phenol, it varied from 62.43 to 61.77%, and for 2,4-DCP, it dropped from 86.53 to 85.95% over CEAC. The difference in removal efficiency significantly remained unchanged for four successive adsorption–desorption cycles. However, a negligible drop in the removal efficiency was also noted. This drop could be due to the sites occupied by phenol/2,4-DCP molecules blocking the pores with an increase in the number of adsorption–desorption cycles.^84^ Further, it was also noted that the desorption efficacy was >95% for all cycles. This higher efficiency was due to the presence of the mesopore structure on CEAC, thus enabling quick removal of the pollutant and creating vacant sites for subsequent cycles. This would also improve the reusability of CEAC and reduce its cost.^85^

#### 3.3.3 Analysis of Adsorption Kinetics of Phenol and 2,4-DCP Removal

**Figure 7:**
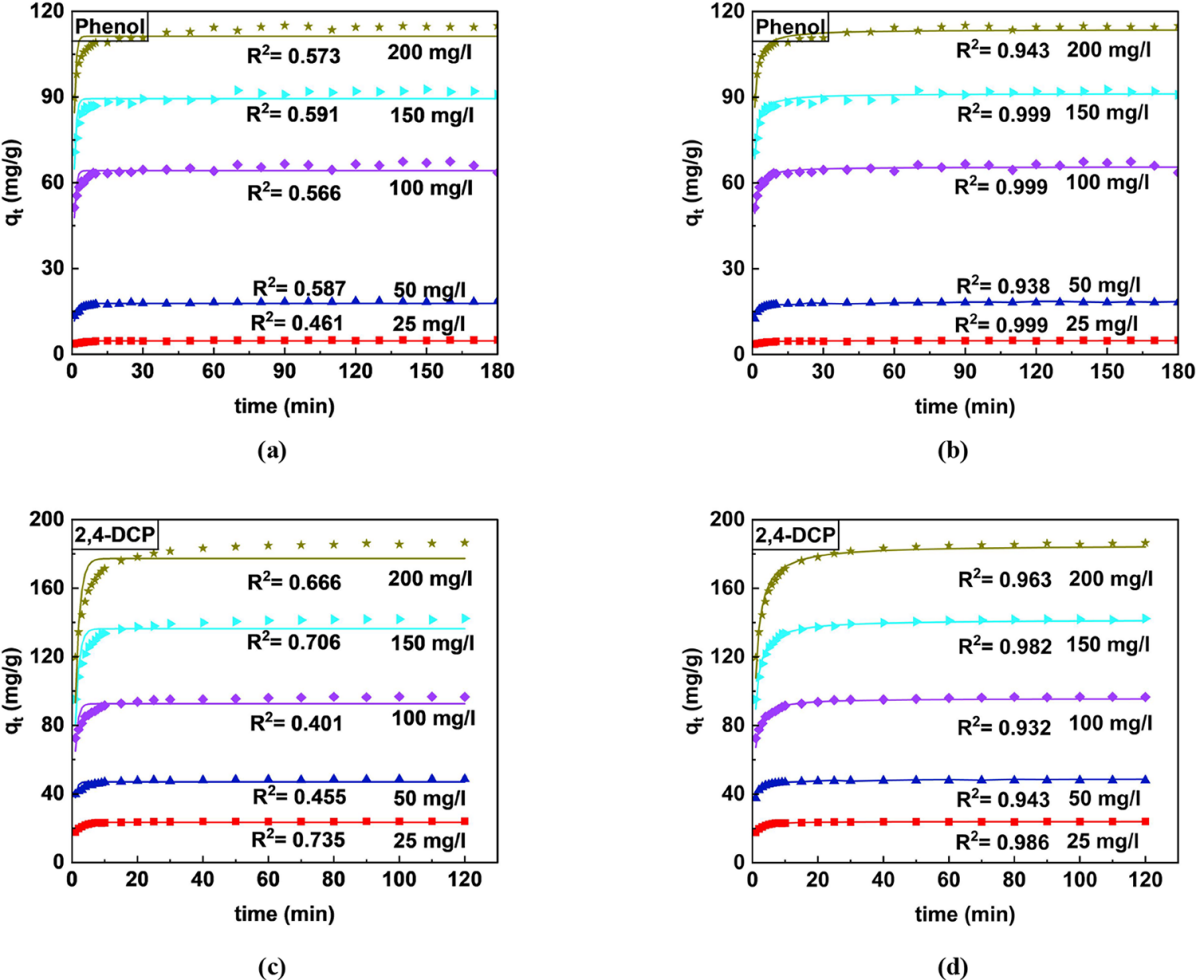
Nonlinear plot of kinetic studies for the adsorption of phenol and 2,4-DCP: (a,c) PFO and (b,d) PSO.

Herein, the adsorption kinetics of phenol and 2,4-DCP at various concentrations (25–200 mg/L) was investigated. The study incorporated the application of PFO and PSO onto experimental data and examined the best model fit among the mentioned one.^45^ The graphical representation of the two models is illustrated in Figure 7. The parameters and constants of these models are listed in Table 5. The high coefficient of determination (*R*^2^) and lower MAPE indicated that the PSO model fits the data better than the PFO model for both phenol and 2,4-DCP, suggesting that chemisorption is the dominant process.^10,55^ From Table 5 as well, the experimental adsorption capacity and projected values by the kinetic model (PSO) showed a considerable match, indicating a strong fit.

**Table 5:** Kinetic Model Parameters for the Adsorption of Phenol and 2,4-DCP on CEAC

		phenol concentration (mg/L)					2,4-DCP concentration (mg/L)				
kinetic models	parameters	25	50	100	150	200	25	50	100	150	200
PFO	*q*_exp_	4.99	18.49	63.56	90.83	114.93	24.07	48.65	96.58	142.32	186.45
	*q*_m_	4.67	17.77	64.23	89.432	111.28	23.367	46.98	92.63	136.37	177.34
	*k*_1_	1.064	1.064	1.361	1.289	1.426	1.174	1.638	1.211	0.887	0.745
	*R*^2^	0.461	0.587	0.566	0.591	0.573	0.735	0.455	0.401	0.706	0.666
	MAPE	0.05	0.04	0.03	0.032	0.031	0.029	0.033	0.045	0.044	0.056
PSO	*q*_m_	4.821	18.25	63.62	91.301	113.64	24.04	48.18	95.79	141.68	185.26
	*k*_2_	0.411	0.119	0.0461	0.0328	0.028	0.108	0.075	0.025	0.012	0.007
	*R*^2^	0.999	0.938	0.999	0.999	0.943	0.986	0.943	0.932	0.982	0.963^a^
	MAPE	0.024	0.013	0.014	0.015	0.011	0.006	0.013	0.015	0.009	0.016

a PSO showed greater fit compared.

The study also states that approximately 70% of the optimum phenol concentration was adsorbed over a span of 10 min, and it took approximately 180 min to reach a state of equilibrium (Figure 7a). For 2,4-DCP, about 90% removal was achieved in less than 10 min, and equilibrium was reached in 120 min (Figure 7b). The recorded timings were less in comparison to the previous studies that were reported.^32,75,86^ The nonlinear kinetic plots for 2,4-DCP are provided and shown in Figure 7c,d. These findings provide valuable insights into the adsorption behavior of these pollutants and can guide the design of effective treatment processes.

#### 3.3.4 Adsorption Thermodynamics

**Figure 8:**
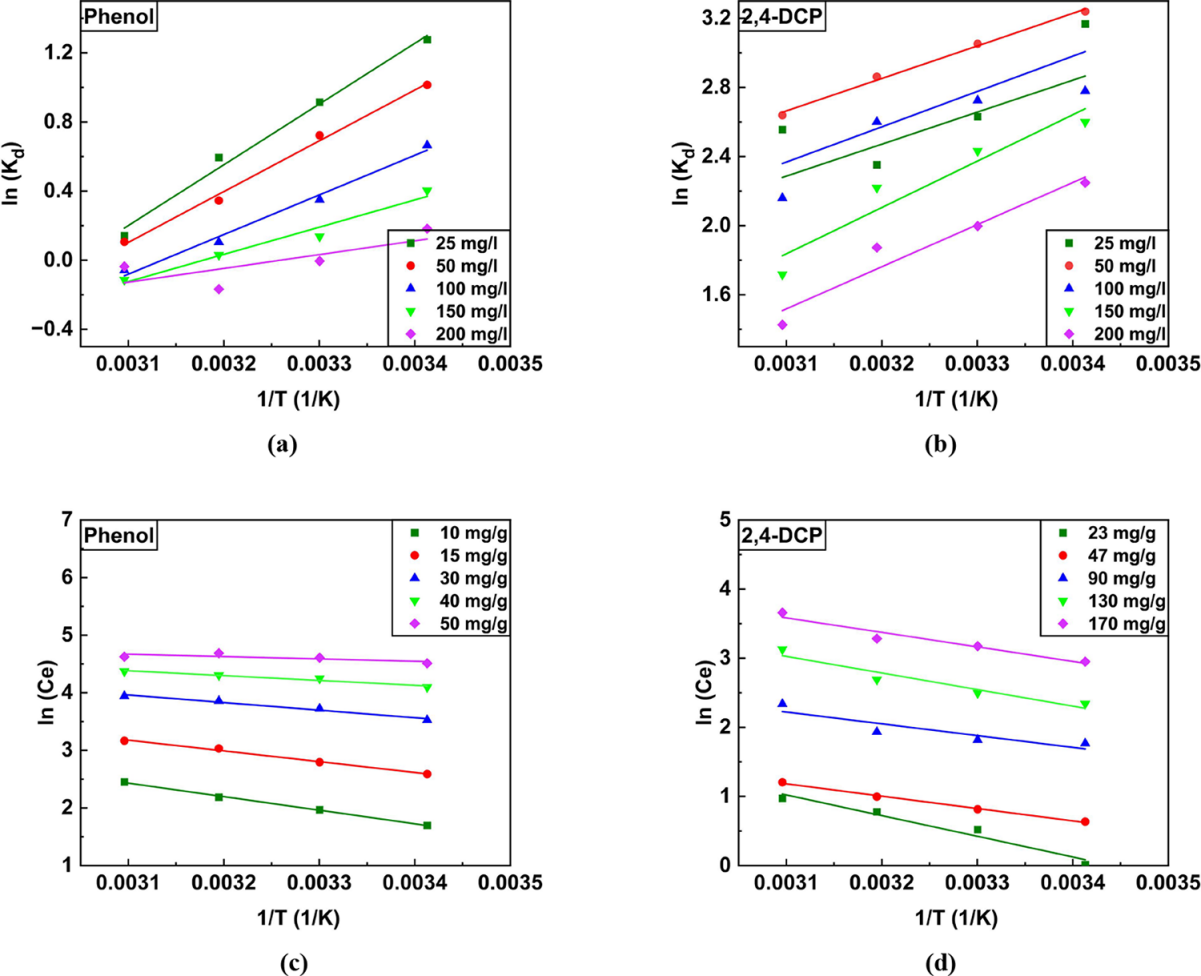
van’t Hoff plot for phenol (a) and 2,4-DCP (b). plot of 1/T versus ln (*C*_e_) for phenol (c) and 2,4-DCP (d).

The assessment of the thermodynamic properties of phenol/2,4-DCP adsorption onto CEAC was performed at different temperatures. The plots of 1/*T* against ln (*K*_d_) depicted in Figure 8a,b are commonly followed in thermodynamics to evaluate the adsorption nature of the pollutant onto the adsorbent by quantifying key thermodynamic parameters. The parameters such as Gibb’s free energy change (Δ*G*^o^), enthalpy change (Δ*H*^o^), and entropy change (Δ*S*^o^) were computed and are listed in Table 6. The negative Δ*G*^o^ values for phenol and 2,4-DCP values obtained for temperatures ranging from 293 to 323 K varied between −3.107 and −0.381 kJ/mol and −7.889 and −7.085 kJ/mol indicate the spontaneous nature of the phenol/2,4-DCP adsorption process. The spontaneity in the adsorption process occurs when the rate of adsorption is greater than the rate of desorption. The dominant adsorption process could be physical if Δ*G*^o^ < −20 kJ/mol or chemical adsorption if Δ*G*^o^ > −40 kJ/mol. Herein, the negative value depicts that the adsorption of both pollutants was mainly influenced by physisorption (Δ*G*^o^ < −20 kJ/mol).^87^ Furthermore, for both pollutants, the negative Δ*H*^o^ values, i.e., −29.22 kJ/mol and −26.70 kJ/mol, corroborate that the process was exothermic, requiring less energy.^34^

**Table 6:** Thermodynamic Data for the Adsorption of Phenol and 2,4-DCP on CEAC

		Δ*G*^o^ (kJ/mol)						
phenolic pollutant	*C*_o_ (mg/L)	283 K	293 K	303 K	313 K	Δ*H*^o^ (kJ/mol)	Δ*S*^o^ (kJ/mol K)	Δ*H*_x_ (kJ/mol)
phenol	25	–3.107	–2.303	–1.545	–0.381	–29.22	–0.089	19.53
2,4-DCP	50	–7.889	–7.689	–7.446	–7.085	–26.70	–0.065	24.86

A negative Δ*S*^o^ implies a decline in entropy or disorder during the adsorption process, while a positive Δ*S*^o^ indicates an increase in entropy or greater randomness.^66^ The entropy change (Δ*S*^o^), with a value < 0.065 kJ/mol, offers insights into the level of disorder or randomness in the thermodynamic system being analyzed. In this context, the negative Δ*S*^o^ value implies that the system experiences a drop in disorder or the molecules become less randomly distributed during the adsorption of phenol/2,4-DCP.^17^

On the other hand, the amount of heat required/released during the adsorption of both pollutants was quantified by plotting ln (*C*_e_) versus 1/*T* in plotted Figure 8b. It can also be termed as heat of adsorption (Δ*H*_x_, kJ/mol). The foremost adsorption process, which is primary, can be concluded based on the value of Δ*H*_x_. The adsorption process can be influenced by physisorption if Δ*H*_x_ < 80 kJ/mol and chemisorption if Δ*H*_x_ > 80 kJ/mol and < 400 kJ/mol. Herein, it was observed that Δ*H*_x_ varied inversely with an increase in the pollutant load (mg/g). For the adsorption of phenol onto CEAC, the heat of adsorption was reduced from 19.53 to 3.37 kJ/mol for an adsorption load ranging from 10 to 50 mg/g. Similarly, the value of Δ*H*_x_ dropped to 14.19 kJ/mol from 24.86 kJ/mol for 2,4-DCP loading varying between 23 and 170 mg/g. Therefore, it can be concluded that the dominant mechanism in the adsorption of all concentrations of phenol and 2,4-DCP was physisorption.^88^ The fall in Δ*H*_x_ was due to the increase in the adsorbate–adsorbate interaction, which was lower at the initial surface loading.

## 4 Plausible Adsorption Mechanism of Phenols

**Figure 9:**
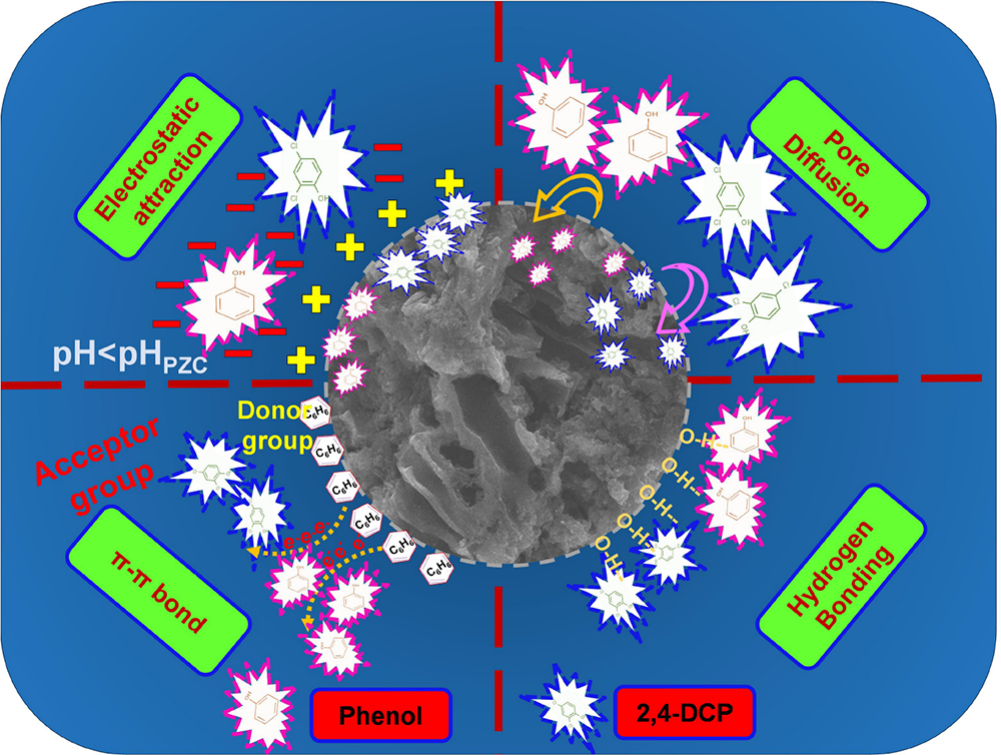
Schematic representation of the adsorption mechanisms for the removal of phenol and 2,4-DCP.

The different mechanisms that could likely occur during the process of adsorption of phenol/2,4-DCP can be explained from characterization, isotherms, kinetics, and thermodynamics. They are pore diffusion, hydrogen bonding, electrostatic attraction (van der Waals forces), physisorption, and chemisorption. A detailed representation of the possible mechanism is shown in Figure 9.

The characterization parameters, such as surface area, pore size, and functional groups, provide essential information about the surface properties. In this regard, it could be said that pore diffusion due to higher surface area and pore size with tubular channels enhanced the adsorption of phenol and 2,4-DCP by providing ample active sites and accommodating pollutant molecules.

The pre- and post-adsorption FTIR spectra are shown in Figure 2b with the presence of an aromatic structure at ∼1600 cm^–1^. These peaks are mainly due to aldehydes, ketones, and carboxylic acids. Functional group such as C=O contribute to phenol/2,4-DCP adsorption through π–π interactions. Herein, the aromatic structure of CEAC interacts with aromatic rings of phenol/2,4-DCP and thus facilitates electron donors and acceptors.^17^

In Figure 2b the presence of an intense peak at approximately ∼3400 cm^–1^ is an indication of hydroxyl functional groups (O–H or N–H). These observed peaks demonstrate that hydrogen bonding between CEAC and phenol molecules might be viable.^88,89^

The shift in these peaks and the drop in the intensity post adsorption depicts the involvement of hydrogen bonding. Further, from the investigation, it was found that the pH of the solution had a significant impact on the adsorption of both pollutants. This was due to the protonating effect that occurred at lower/acidic pH < 3.89. Thus, it can be concluded that electrostatic type (at pH < pH_PZC_) of attraction has also played an important role in the adsorption of these pollutants.^90^

Furthermore, from the isotherm models, it can be noted that most of them fitted better with *R*^2^ > 0.99. From the DR model, three different types of adsorptions can be proposed based on the free energy values such as physical adsorption (*E* < 8 kJ/mol), electrostatic adsorption (8 < *E* < 16 kJ/mol), and chemical adsorption (*E* > 16 kJ/mol). Herein, the better fit of the adsorption model and adsorption energy values suggests that phenol (*E* = 235.7 kJ/mol) and 2,4-DCP (*E* = 845.17 kJ/mol) removal might have followed chemical adsorption.^91^

## 5 Conclusions

This study employed activated carbon derived from *Casuarina equisetifolia* seeds, a form of agricultural waste, using a relatively low activation temperature of 773 K. The end product, i.e., activated carbon, exhibited an extensive amorphous and porous structure with an outstanding surface area of 1007 m^2^/g and a pore volume of 0.6521 m^3^/g, classifying it as mesoporous carbon. The SEM-EDS analysis of the developed adsorbent unveiled a highly porous and predominant carbon composition (81.78%).

It is noteworthy that it showcases a remarkable adsorption performance, with the adsorbent showcasing a 76% removal efficiency for phenol and a 92% efficiency for 2,4-DCP. Additionally, the adsorption capacities were substantial, reaching 364.62 mg/g for phenol and 382.03 mg/g for 2,4-DCP at a pH of 2. Likewise, analysis of isotherm data suggested a better fit to the Freundlich and Langmuir model for phenol and 2,4-DCP, exhibiting *R*^2^ values above 0.99.

Kinetic investigations for both pollutants emphasized the predominance of PSO kinetics with *R*^2^ > 0.999 and MAPE < 0.024, implying the major involvement of chemisorption in pollutant uptake. Furthermore, it is noteworthy to mention that the adsorptive removal of phenol (50%) and 2,4-DCP (85%) was witnessed in less than 30 min.

Thermodynamic analysis uncovered an extremely lower Δ*H*^o^ < 0 with a magnitude below 25 kJ/mol, implying the significance of physical adsorption and exothermic process. The obtained enthalpy change values were consistent with the isosteric heat of adsorption (Δ*H*_x_, kJ/mol) in the case of phenol and 2,4-DCP. Thus, this corroborates the dominance of physisorption throughout the adsorption. Further, from the desorption studies, a consistent performance of activated carbon was observed for four cycles. In addition, the dominant mechanism was found to be pore diffusion and electrostatic attraction coupled with π–π interaction and hydrogen bonding. The overall results underline the potential of *Casuarina equisetifolia* seeds as an environmentally friendly resource to produce mesoporous activated carbon, suggesting a substitute for pollutant mitigation.

## Supporting Information Available

Steps for the calculation of adsorption isotherm parameters and constants in the DR model; steps for the calculation of adsorption kinetic parameters and constants; steps for the calculation of thermodynamic parameters (Δ*H*^o^, Δ*G*^o^, and Δ*S*^o^); and steps for the calculation of thermodynamic parameters (Δ*H*_x_) (PDF)

## Glossary

*C*_o_: initial concentration of pollutant (mg/L)
*C*_e_: concentration of pollutant at equilibrium (mg/L)
*C*_a_: concentration of pollutant in solid phase (mg/L)
*C*_d_: concentration of pollutant in eluent (mg/L)
*V*: volume of pollutant (mL)
*m*: amount of adsorbent (mg)
*q*_e_: experimental adsorption capacity (mg/g)
*q*_m_: monolayer adsorption capacity (mg/g)
*K*_L_: Langmuir constant (L/mg)
*K*_F_: Freundlich constant (L/g)
*n*: heterogeneity coefficient (dimensionless)
*b*: Temkin binding energy (J/mol)
*A*_T_: equilibrium bonding constant (L/g)
*K*_RP_: Redlich Peterson constant (L/mg)
β: Redlich Peterson constant (dimensionless)
*a*_RP_: Redlich Peterson constant (L/mg)
*k*_ad_: activity coefficient (mol^2^/kJ^2^)
ε: polyani exponential (kJ/mol)
*E*: adsorption energy (kJ/mol)
*q_t_*: experimental adsorption capacity at time “*t*” (mg/g)
*k*_1_: pseudo-first-order rate constant (1/min)
*k*_2_: pseudo-second-order rate constant (g/mg/min)
Δ*S*^o^: entropy change (kJ/mol/K)
Δ*H*^o^: enthalpy change (kJ/mol)
Δ*G*^o^: Gibbs free energy change (kJ/mol)
Δ*H*_x_: isosteric heat of adsorption (kJ/mol)

## References

[ref1] Dehmani Y.; Lamhasni T.; Mohsine A.; Tahri Y.; Lee H. S.; Lgaz H.; Alrashdi A. A.; Abouarnadasse S. Adsorption Removal of Phenol by Oak Wood Charcoal Activated Carbon. Biomass Convers. Biorefin. 2022, 14, 8015–8027. 10.1007/s13399-022-03036-5.

[ref2] Sathishkumar M.; Binupriya A. R.; Kavitha D.; Selvakumar R.; Jayabalan R.; Choi J. G.; Yun S. E. Adsorption Potential of Maize Cob Carbon for 2,4-Dichlorophenol Removal from Aqueous Solutions: Equilibrium, Kinetics and Thermodynamics Modeling. Chem. Eng. J. 2009, 147 (2–3), 265–271. 10.1016/j.cej.2008.07.020.

[ref3] Liu Q.; Yang B.; Zhang L.; Huang R. Simultaneous Adsorption of Phenol and Cu2+ from Aqueous Solution by Activated Carbon/Chitosan Composite. Korean J. Chem. Eng. 2014, 31 (9), 1608–1615. 10.1007/s11814-014-0080-9.

[ref4] Singh N.; Agarwal B.; Balomajumder C. Simultaneous Treatment of Phenol and Cyanide Containing Aqueous Solution by Adsorption, Biotreatment and Simultaneous Adsorption and Biotreatment (SAB) Process. J. Environ. Chem. Eng. 2016, 4 (1), 564–575. 10.1016/j.jece.2015.11.041.

[ref5] Ghahghaey Z.; Hekmati M.; Darvish Ganji M. Theoretical Investigation of Phenol Adsorption on Functionalized Graphene Using DFT Calculations for Effective Removal of Organic Contaminants from Wastewater. J. Mol. Liq. 2021, 324, 114777 10.1016/j.molliq.2020.114777.

[ref6] Mohammadi S. Z.; Darijani Z.; Karimi M. A. Fast and Efficient Removal of Phenol by Magnetic Activated Carbon-Cobalt Nanoparticles. J. Alloys Compd. 2020, 832, 154942 10.1016/j.jallcom.2020.154942. 33031063

[ref7] Sun J.; Liu X.; Zhang F.; Zhou J.; Wu J.; et al. Insight into the Mechanism of Adsorption of Phenol and Resorcinol on Activated Carbons with Different Oxidation Degrees. Colloids Surf., A 2019, 563, 22–30. 10.1016/j.colsurfa.2018.11.042.

[ref8] Kulkarni S. J; Kaware J. P. Review on Research for Removal of Phenol from Wastewater. Int. J. Sci. Res. Publ. 2013, 3 (4), 1–5. 10.70729/J20138.

[ref9] Yu L.; Gamliel D. P.; Markunas B.; Valla J. A. A Promising Solution for Food Waste: Preparing Activated Carbons for Phenol Removal from Water Streams. ACS Omega 2021, 6 (13), 8870–8883. 10.1021/acsomega.0c06029. 33842758 PMC8028020

[ref10] Mishra S.; Yadav S. S.; Rawat S.; Singh J.; Koduru J. R. Corn Husk Derived Magnetized Activated Carbon for the Removal of Phenol and Para-Nitrophenol from Aqueous Solution: Interaction Mechanism, Insights on Adsorbent Characteristics, and Isothermal, Kinetic and Thermodynamic Properties. J. Environ. Manage. 2019, 246, 362–373. 10.1016/j.jenvman.2019.06.013. 31195256

[ref11] Wahid S. N.; Maharaj R.; Boodlal D.; Smith J. V. The Adsorption of Phenol on Granular Activated Carbon Prepared from Waste Coconut Shell in Trinidad. Environ. Prog. Sustainable Energy 2022, 41 (1), e13729 10.1002/ep.13729.

[ref12] Sathya Priya D.; Sureshkumar M. V. Synthesis of Borassus Flabellifer Fruit Husk Activated Carbon Filter for Phenol Removal from Wastewater. Int. J. Environ. Sci. Technol. 2020, 17 (2), 829–842. 10.1007/s13762-019-02325-3.

[ref13] Medellín-Castillo N. A.; Ocampo-Pérez R.; Forgionny A.; Labrada-Delgado G. J.; Zárate-Guzmán A. I.; et al. Insights into Equilibrium and Adsorption Rate of Phenol on Activated Carbon Pellets Derived from Cigarette Butts. Processes 2021, 9 (6), 934 10.3390/pr9060934.

[ref14] Huang K.; Yang S.; Liu X.; Zhu C.; Qi F.; et al. Adsorption of Antibiotics from Wastewater by Cabbage-Based N, P Co-Doped Mesoporous Carbon Materials. J. Clean. Prod. 2023, 391, 136174 10.1016/j.jclepro.2023.136174.

[ref15] Kuśmierek K.; Świątkowski A. Adsorption of Phenols on Carbonaceous Materials of Various Origins but of Similar Specific Surface Areas. Separations 2023, 10 (8), 422 10.3390/separations10080422.

[ref16] Vohra M.; Hussaini M.; Mohammad T. Olive Branches Activated Carbon: Synthesis. Phenol Adsorption and Modeling. Chem. Pap. 2023, 77 (1), 485–498. 10.1007/s11696-022-02457-w.

[ref17] Cho E. J.; Lee C. G.; Kang J.-K.; Park S. J. Adsorption of Phenol on Kenaf-derived Biochar: Studies on Physicochemical and Adsorption Characteristics and Mechanism. Biomass Convers. Biorefin. 2024, 14, 9621–9638. 10.1007/s13399-022-03262-x.

[ref18] Gama B. M. V.; Sales D. C. S.; Nascimento G. E.; Rodriguez-Díaz J. M.; Barbosa C. M. B. M.; Duarte M. M. M. B. Modeling Mono- and Multicomponent Adsorption of Phenol and Cadmium from Aqueous Solution by Peanut Shell Biochar. Ind. Eng. Chem. Res. 2022, 61 (51), 18833–18842. 10.1021/acs.iecr.2c02618.

[ref19] Nagarajan L.; Saravanan P.; Kumaraguru K.; AnnamRenita A.; Rajeshkannan R.; Rajasimman M. A Facile Approach in Activated Carbon Synthesis from Wild Sugarcane for Carbon Dioxide Capture and Recovery: Isotherm and Kinetic Studies. Biomass Convers. Biorefin. 2024, 14, 9595–9607. 10.1007/s13399-022-03080-1.

[ref20] Yahya M. D.; Abubakar H.; Obayomi K. S.; Iyaka Y. A.; Suleiman B. Simultaneous and Continuous Biosorption of Cr and Cu (II) Ions from Industrial Tannery Effluent Using Almond Shell in a Fixed Bed Column. Results Eng. 2020, 6 (March), 100113 10.1016/j.rineng.2020.100113.

[ref21] Xia Y.; Zuo H.; Lv J.; Wei S.; Yao Y.; Liu Z.; Lin Q.; Yu Y.; Yu W.; Huang Y. Preparation of Multi-Layered Microcapsule-Shaped Activated Biomass Carbon with Ultrahigh Surface Area from Bamboo Parenchyma Cells for Energy Storage and Cationic Dyes Removal. J. Clean. Prod. 2023, 396, 136517 10.1016/j.jclepro.2023.136517.

[ref22] Abdallah W. E.; Shams K. A.; El-Shamy A. M. Phytochemical Analysis and Evaluation of Its Antioxidant, Antimicrobial, and Cytotoxic Activities for Different Extracts of Casuarina Equisetifolia. BMC Complement. Med. Ther. 2024, 24 (1), 128 10.1186/s12906-024-04422-4. 38509538 PMC10956242

[ref23] Yang Z.; Zhao Z.; Yang X.; Ren Z. Xanthate Modified Magnetic Activated Carbon for Efficient Removal of Cationic Dyes and Tetracycline Hydrochloride from Aqueous Solutions. Colloids Surfaces A Physicochem. Eng. Asp. 2021, 615, 126273 10.1016/j.colsurfa.2021.126273.

[ref24] Chandarana H.; Suganya S.; Madhava A. K. Surface Functionalized Casuarina Equisetifolia Pine Powder for the Removal of Hetero-Polyaromatic Dye: Characteristics and Adsorption. Int. J. Environ. Anal. Chem. 2022, 102 (17), 5457–5471. 10.1080/03067319.2020.1798418.

[ref25] Chandarana H.; Senthil Kumar P.; Seenuvasan M.; Anil Kumar M. Kinetics, Equilibrium and Thermodynamic Investigations of Methylene Blue Dye Removal Using Casuarina Equisetifolia Pines. Chemosphere 2021, 285 (May), 131480 10.1016/j.chemosphere.2021.131480. 34265726

[ref26] Ravichandran P.; Sugumaran P.; Seshadri S.; Basta A. H. Optimizing the Route for Production of Activated Carbon from Casuarina Equisetifolia Fruit Waste. R. Soc. Open Sci. 2018, 5 (7), 171578 10.1098/rsos.171578. 30109042 PMC6083678

[ref27] Zuki A. A. A.; Awang M.; Mahmud A. A.; Jaafar J. J.; Zain M. H. Removal of Neutral Red Dye from Aqueous Solution by Raw and Microwave-Chemical Modified Coastal Plant, Casuarina Equisetifolia Seeds as Adsorbents. Int. J. Appl. Chem. 2016, 12 (1), 29–33.

[ref28] Dahri M. K.; Kooh M. R. R.; Lim L. B. L. Application of Casuarina Equisetifolia Needle for the Removal of Methylene Blue and Malachite Green Dyes from Aqueous Solution. Alexandria Eng. J. 2015, 54 (4), 1253–1263. 10.1016/j.aej.2015.07.005.

[ref29] Mohan S.; Sumitha K. Removal of Cu (II) by Adsorption Using Casuarina Equisetifolia Bark. Environ. Eng. Sci. 2008, 25 (4), 497–506. 10.1089/ees.2006.0221.

[ref30] El Nemr A.; El Sikaily A.; Khaled A.; Abdelwahab O. Removal of Toxic Chromium(VI) from Aqueous Solution by Activated Carbon Using Casuarina Equisetifolia. Chem. Ecol. 2007, 23 (2), 119–129. 10.1080/02757540701197754.

[ref31] Ranganathan K. Chromium Removal by Activated Carbons Prepared from Casurina Equisetifolia Leaves. Bioresour. Technol. 2000, 73 (2), 99–103. 10.1016/S0960-8524(99)00176-5.

[ref32] Dehmani Y.; Franco D. S. P.; Georgin J.; Lamhasni T.; Brahmi Y.; et al. Towards Experimental and Theoretical Understanding of the Adsorption Behavior of Phenol on a New Activated Carbon Prepared from Oak Wood. J. Water Process Eng. 2023, 54, 103936 10.1016/j.jwpe.2023.103936.

[ref33] Zhang G.; Zhou L.; Chi T.; Fan X.; Fang Y.; Zou H.; et al. Effect of Pyrolytic Temperatures on the 2,4-Dichlorophenol Adsorption Performance of Biochar Derived from Populus Nigra. Environ. Sci. Pollut. Res. 2024, 1–11. 10.1007/s11356-024-31990-2. 38236571

[ref34] Patil P.; Jeppu G.; Vallabha M. S.; Girish C. R. Enhanced Adsorption of Phenolic Compounds Using Biomass-Derived High Surface Area Activated Carbon: Isotherms, Kinetics and Thermodynamics. Environ. Sci. Pollut. Res. 2024, 31, 67442 10.1007/s11356-024-32971-1. PMC11685270 38578594

[ref35] Tao J.; Huo P.; Fu Z.; Zhang J.; Yang Z.; Zhang D. Characterization and Phenol Adsorption Performance of Activated Carbon Prepared from Tea Residue by NaOH Activation. Environ. Technol. 2019, 40 (2), 171–181. 10.1080/09593330.2017.1384069. 28934911

[ref36] Okeowo I. O.; Balogun E. O.; Ademola A. J.; Alade A. O.; Afolabi T. J.; et al. Adsorption of Phenol from Wastewater Using Microwave-Assisted Ag–Au Nanoparticle-Modified Mango Seed Shell-Activated Carbon. Int. J. Environ. Res. 2020, 14 (2), 215–233. 10.1007/s41742-020-00244-7.

[ref37] Nwabanne J. T.; Iheanacho O. C.; Obi C. C.; Onu C. E. Linear and Nonlinear Kinetics Analysis and Adsorption Characteristics of Packed Bed Column for Phenol Removal Using Rice Husk-Activated Carbon. Appl. Water Sci. 2022, 12 (5), 1–16. 10.1007/s13201-022-01635-1.

[ref39] Li Z.; Mao Y.; Liu Z.; Song Z.; et al. Efficient Adsorption and Removal Mechanism of 2,4-Dichlorophenol by MoS2@C6H12O6 Floral Activated Carbon with Intercalated Structure. Mater. Sci. Eng., B 2024, 299, 116807 10.1016/j.mseb.2023.116807.

[ref40] Nirmala G.; Murugesan T.; Rambabu K.; Sathiyanarayanan K.; Show P. L. Adsorptive Removal of Phenol Using Banyan Root Activated Carbon. Chem. Eng. Commun. 2021, 208 (6), 831–842. 10.1080/00986445.2019.1674839.

[ref41] Paranjape P.; Sadgir P. Linear and Nonlinear Regression Methods for Isotherm and Kinetic Modelling of Iron Ions Bioadsorption Using Ocimum Sanctum Linn. Leaves from Aqueous Solution. Water Pract. Technol. 2023, 18 (8), 1807–1827. 10.2166/wpt.2023.110.

[ref42] Pei T.; Shi F.; Liu C.; Lu Y.; Lin X.; et al. Bamboo-Derived Nitrogen-Doping Magnetic Porous Hydrochar Coactivated by K_2_FeO_4_ and CaCO_3_ for Phenol Removal: Governing Factors and Mechanisms. Environ. Pollut. 2023, 331, 121871 10.1016/j.envpol.2023.121871. 37225081

[ref43] Fathy M. A.; Kamel A. H.; Hassan S. S. M. Novel Magnetic Nickel Ferrite Nanoparticles Modified with Poly(Aniline- Co-o -Toluidine) for the Removal of Hazardous 2,4-Dichlorophenol Pollutant from Aqueous Solutions. RSC Adv. 2022, 12 (12), 7433–7445. 10.1039/D2RA00034B. 35424706 PMC8982154

[ref44] Kilic M.; Apaydin-Varol E.; Pütün A. E. Adsorptive Removal of Phenol from Aqueous Solutions on Activated Carbon Prepared from Tobacco Residues: Equilibrium, Kinetics and Thermodynamics. J. Hazard. Mater. 2011, 189 (1–2), 397–403. 10.1016/j.jhazmat.2011.02.051. 21420235

[ref45] Patil P.; Jeppu G.; Girish C. R.; Mohan B. Development of a Comprehensive Analytical Solution for Modeling Adsorption Kinetics and Equilibrium. Sep. Sci. Technol. 2024, 59 (3), 373–394. 10.1080/01496395.2024.2319146.

[ref46] Anumasahun O. V.; Akinola A. O.; Bello O. O.; Agboola O. S.; Bello O. S. Removal of 2,4-Dichlorophenol from Aqueous Medium Using Activated Carbon Prepared from Cocoa Pod Husks. Chem. Data Collect. 2023, 44, 100997 10.1016/j.cdc.2023.100997.

[ref47] Kunwar B.; Mondal S.; Saini V. K.; Bahukhandi K. D.; Kumar A. Utilization of Barks of Araucaria Columnaris: Preparation of Activated Carbon/Clay Composite Beads and Adsorptive Treatment of Phenolic Wastewater. Ind. Crops Prod. 2023, 197, 116534 10.1016/j.indcrop.2023.116534.

[ref48] Singh Y.; Kumar M.; Kumar A. Removal of Phenol from Aqueous Solution by Mahua Seed Activated Carbon: Kinetic, Isotherm, Mass Transfer and Isosteric Heat of Adsorption Studies. Chem. Ind. Chem. Eng. Q. 2016, 22 (3), 263–273. 10.2298/CICEQ150327040S.

[ref49] Neme I.; Gonfa G.; Masi C. Activated Carbon from Biomass Precursors Using Phosphoric Acid: A Review. Heliyon 2022, 8 (12), e11940 10.1016/j.heliyon.2022.e11940. 36478849 PMC9720030

[ref50] Li Y.; Zhang X.; Yang R.; Li G.; Hu C. The Role of H3PO4 in the Preparation of Activated Carbon from NaOH-Treated Rice Husk Residue. RSC Adv. 2015, 5 (41), 32626–32636. 10.1039/C5RA04634C.

[ref51] Neme I.; Gonfa G.; Masi C. Preparation and Characterization of Activated Carbon from Castor Seed Hull by Chemical Activation with H3PO4. Results Mater. 2022, 15 (July), 100304 10.1016/j.rinma.2022.100304.

[ref52] Raut E. R.; Bedmohata M. A.; Chaudhari A. R. Comparative Study of Preparation and Characterization of Activated Carbon Obtained from Sugarcane Bagasse and Rice Husk by Using H3PO4 and ZnCl2. Mater. Today Proc. 2022, 66, 1875–1884. 10.1016/j.matpr.2022.05.413.

[ref53] Danish M.; Pin Z.; Ziyang L.; Ahmad T.; Majeed S.; Yahya A. N.; et al. Preparation and Characterization of Banana Trunk Activated Carbon Using H_3_PO_4_ Activation: A Rotatable Central Composite Design Approach. Mater. Chem. Phys. 2022, 282, 125989 10.1016/j.matchemphys.2022.125989.

[ref54] Franco D. S. P.; Georgin J.; Netto M. S.; Allasia D.; Oliveira M. L. S.; Foletto E. L.; Dotto G. L. Highly Effective Adsorption of Synthetic Phenol Effluent by a Novel Activated Carbon Prepared from Fruit Wastes of the Ceiba Speciosa Forest Species. J. Environ. Chem. Eng. 2021, 9 (5), 105927 10.1016/j.jece.2021.105927.

[ref55] Wang W.; Wang Z.; Li K.; Liu Y.; Xie D.; Shan S.; et al. Enhanced Adsorption of Aqueous Chlorinated Aromatic Compounds by Nitrogen Auto-Doped Biochar Produced through Pyrolysis of Rubber-Seed Shell. Environ. Technol. 2023, 44 (5), 631–646. 10.1080/09593330.2021.1980829. 34516358

[ref56] Aravindhan S.; Kumar G. B.; Saravanan M.; Arumugam A. Delonix Regia Biomass as an Eco-Friendly Biosorbent for Effective Alizarin Red S Textile Dye Removal: Characterization, Kinetics, and Isotherm Studies. Bioresour. Technol. Rep. 2024, 25, 101721 10.1016/j.biteb.2023.101721.

[ref57] Xie B.; Qin J.; Wang S.; Li X.; Sun H.; Chen W. Adsorption of Phenol on Commercial Activated Carbons: Modelling and Interpretation. Int. J. Environ. Res. Public Health 2020, 17 (3), 789 10.3390/ijerph17030789. 32012816 PMC7037044

[ref58] Braghiroli F. L.; Bouafif H.; Hamza N.; Neculita C. M.; Koubaa A. Production, Characterization, and Potential of Activated Biochar as Adsorbent for Phenolic Compounds from Leachates in a Lumber Industry Site. Environ. Sci. Pollut. Res. 2018, 25 (26), 26562–26575. 10.1007/s11356-018-2712-9. 29992415

[ref59] Vinayagam R.; Kar A.; Murugesan G.; Varadavenkatesan T.; Goveas L. C.; Samanth A.; Ahmed M. B.; Selvaraj R. Low Temperature Carbonized Mesoporous Graphitic Carbon for Tetracycline Adsorption: Mechanistic Insight and Adaptive Neuro-Fuzzy Inference System Modeling. Bioresour. Technol. Rep. 2023, 22, 101468 10.1016/j.biteb.2023.101468.

[ref60] Luo Y.; Li D.; Chen Y.; Sun X.; Cao Q.; Liu X. The Performance of Phosphoric Acid in the Preparation of Activated Carbon-Containing Phosphorus Species from Rice Husk Residue. J. Mater. Sci. 2019, 54 (6), 5008–5021. 10.1007/s10853-018-03220-x.

[ref61] Ezung S. L.; Baruah M.; Supong A.; Sharma S.; Sinha D. Experimental and Theoretical Insight into the Adsorption of 2,4-Dichlorophenol on Low-Cost Bamboo Sheath Activated Carbon. Sustainable Chem. Pharm. 2022, 26, 100643 10.1016/j.scp.2022.100643.

[ref62] Zhao M.-H.; Bai X.; Fan X.; Li Y.; Liu Y.; Huang J.-L.; et al. Removal Behaviors of Phenol from Aqueous Solution Using Industrial Coal Sludge-Derived Porous Carbon Sorbent. J. Mol. Liq. 2023, 385, 122427 10.1016/j.molliq.2023.122427.

[ref63] Kong X.; Gao H.; Song X.; Deng Y.; Zhang Y. Adsorption of Phenol on Porous Carbon from Toona Sinensis Leaves and Its Mechanism. Chem. Phys. Lett. 2020, 739, 137046 10.1016/j.cplett.2019.137046.

[ref64] Sriramoju S. K.; Dash P. S.; Majumdar S. Meso-Porous Activated Carbon from Lignite Waste and Its Application in Methylene Blue Adsorption and Coke Plant Effluent Treatment. J. Environ. Chem. Eng. 2021, 9 (1), 104784 10.1016/j.jece.2020.104784.

[ref65] Kumar M.; Tamilarasan R. Modeling of Experimental Data for the Adsorption of Methyl Orange from Aqueous Solution Using a Low Cost Activated Carbon Prepared from Prosopis Julifl Ora. Polish J. Chem. Technol. 2013, 15 (2), 29–39. 10.2478/pjct-2013-0021.

[ref66] Supong A.; Bhomick P. C.; Karmaker R.; Ezung S. L.; Jamir L.; et al. Experimental and Theoretical Insight into the Adsorption of Phenol and 2,4-Dinitrophenol onto Tithonia Diversifolia Activated Carbon. Appl. Surf. Sci. 2020, 529, 147046 10.1016/j.apsusc.2020.147046.

[ref67] Adar E.; Atay I.; Buncu K. G.; Bilgili M. S. Phenol Removal from Synthetic Wastewater with Powdered Activated Carbon: Isotherms, Kinetics and Thermodynamics. Environ. Res. Technol. 2020, 3 (1), 8–14. 10.35208/ert.692302.

[ref68] Darla U. R.; Lataye D. H.; Kumar A.; Pandit B.; Ubaidullah M. Adsorption of Phenol Using Adsorbent Derived from Saccharum Officinarum Biomass: Optimization, Isotherms, Kinetics, and Thermodynamic Study. Sci. Rep. 2023, 13 (1), 1–13. 10.1038/s41598-023-42461-y. 37884549 PMC10603077

[ref69] Ho Z. H.; Adnan L. A. Phenol Removal from Aqueous Solution by Adsorption Technique Using Coconut Shell Activated Carbon. Trop. Aquat. Soil Pollut. 2021, 1 (2), 98–107. 10.53623/tasp.v1i2.21.

[ref70] Song Y.; Wang Y.; Han R. Adsorption of Chlorophenols on Activated Pine Sawdust-Activated Carbon from Solution in Batch Mode. Environ. Sci. Pollut. Res. 2023, 30 (11), 31294–31308. 10.1007/s11356-022-24403-9. 36445525

[ref71] Rambabu K.; Banat F.; Nirmala G. S.; Velu S.; Monash P.; Arthanareeswaran G. Activated Carbon from Date Seeds for Chromium Removal in Aqueous Solution. Desalin. WATER Treat. 2019, 156, 267–277. 10.5004/dwt.2018.23265.

[ref72] Mandal A.; Das S. K. Adsorptive Removal of Phenol by Activated Alumina and Activated Carbon from Coconut Coir and Rice Husk Ash. Water Conserv. Sci. Eng. 2019, 4 (4), 149–161. 10.1007/s41101-019-00075-4.

[ref73] Singh M.; Rayaz M.; Arti R. Isotherm and Kinetic Studies for Sorption of Cr(VI) onto Prosopis Cineraria Leaf Powder: A Comparison of Linear and Non-Linear Regression Analysis. Environ. Prog. Sustainable Energy 2024, 43 (1), e14259 10.1002/ep.14259.

[ref74] Melo J. M.; Lütke S. F.; Igansi A. V.; Franco D. S. P.; Vicenti J. R. M.; et al. Mass Transfer and Equilibrium Modelings of Phenol Adsorption on Activated Carbon from Olive Stone. Colloids Surf., A 2024, 680, 132628 10.1016/j.colsurfa.2023.132628.

[ref75] da Silva M. C. F.; Lütke S. F.; Nascimento V. X.; Lima É. C.; et al. Activated Carbon Prepared from Brazil Nut Shells towards Phenol Removal from Aqueous Solutions. Environ. Sci. Pollut. Res. 2023, 30 (34), 82795–82806. 10.1007/s11356-023-28268-4. 37336851

[ref76] Alam M. G.; Danish M.; Alanazi A. M.; Ahmad T.; HPS A. K. Response Surface Methodology Approach of Phenol Removal Study Using High-Quality Activated Carbon Derived from H_3_PO_4_ Activation of Acacia Mangium Wood. Diamond Relat. Mater. 2023, 132 (312), 109632 10.1016/j.diamond.2022.109632.

[ref77] Abdulrahman M. S.; Alsarayreh A. A.; Barno S. K. A.; Abd Elkawi M. A.; Abbas A. S. Activated Carbon from Sugarcane as an Efficient Adsorbent for Phenol from Petroleum Refinery Wastewater: Equilibrium, Kinetic, and Thermodynamic Study. Open Eng. 2023, 13 (1), 20220442 10.1515/eng-2022-0442.

[ref78] Boughaita I.; Foudia M.; Durand A.; Kherraf S.; Bouchelta C.; Medjram M. S. Phenol Adsorption on Modified Adsorbents NH3-Activated Carbon, Naoh-Activated Carbon: Characterization, Kinetic and Isotherm Modeling. Iran. J. Chem. Chem. Eng. 2023, 42 (5), 1574–1585. 10.30492/ijcce.2022.554065.5346.

[ref79] Asgharnia H.; Nasehinia H.; Rostami R.; Rahmani M.; Mehdinia S. M. Phenol Removal from Aqueous Solution Using Silica and Activated Carbon Derived from Rice Husk. Water Pract. Technol. 2019, 14 (4), 897–907. 10.2166/wpt.2019.072.

[ref80] Ahmed N.; Rahman M. A. Adsorptive Removal of 2,4-Dichlorophenol from Aqueous Solution by Using Used Black Tea Leaves. J. Mex. Chem. Soc. 2021, 65 (2), 225–236. 10.29356/jmcs.v65i2.1424.

[ref81] Alver A.; Baştürk E.; Tulun Ş.; Şimşek İ. Adaptive Neuro-Fuzzy Inference System Modeling of 2,4-Dichlorophenol Adsorption on Wood-Based Activated Carbon. Environ. Prog. Sustain. Energy 2020, 39 (5), 1–12. 10.1002/ep.13413.32832013

[ref82] Ma J. W.; Wang H.; Wang F. Y.; Huang Z. H. Adsorption of 2,4-Dichlorophenol from Aqueous Solution by a New Low-Cost Adsorbent - Activated Bamboo Charcoal. Sep. Sci. Technol. 2010, 45 (16), 2329–2336. 10.1080/01496395.2010.504482.

[ref83] Akhtar M.; Bhanger M. I.; Iqbal S.; Hasany S. M. Sorption Potential of Rice Husk for the Removal of 2,4-Dichlorophenol from Aqueous Solutions: Kinetic and Thermodynamic Investigations. J. Hazard. Mater. 2006, 128 (1), 44–52. 10.1016/j.jhazmat.2005.07.025. 16126338

[ref84] Hu R.; Dai S.; Shao D.; Alsaedi A.; Ahmad B.; Wang X. Efficient Removal of Phenol and Aniline from Aqueous Solutions Using Graphene Oxide/Polypyrrole Composites. J. Mol. Liq. 2015, 203, 80–89. 10.1016/j.molliq.2014.12.046.

[ref85] Shi W.; Wang H.; Yan J.; Shan L.; Quan G.; Pan X.; et al. Wheat Straw Derived Biochar with Hierarchically Porous Structure for Bisphenol A Removal: Preparation, Characterization, and Adsorption Properties. Sep. Purif. Technol. 2022, 289, 120796 10.1016/j.seppur.2022.120796.

[ref86] Channa A. M.; Baytak S.; Memon S. Q.; Talpur M. Y. Equilibrium, Kinetic and Thermodynamic Studies of Removal of Phenol from Aqueous Solution Using Surface Engineered Chemistry. Heliyon 2019, 5 (6), e01852 10.1016/j.heliyon.2019.e01852. 31194060 PMC6551471

[ref87] Almahbashi N. M. Y.; Kutty S. R. M.; Jagaba A. H.; Al-nini A.; Al-Dhawi B. N. S.; Rathnayake U. Phenol Removal from Aqueous Solutions Using Rice Stalk-Derived Activated Carbon: Equilibrium, Kinetics, and Thermodynamics Study. Case Stud. Chem. Environ. Eng. 2023, 8, 100471 10.1016/j.cscee.2023.100471.

[ref88] Iheanacho O. C.; Nwabanne J. T.; Obi C. C.; Igwegbe C. A.; Onu C. E.; Dahlan I. Adsorptive Dephenolization of Aqueous Solutions Using Thermally Modified Corn Cob: Mechanisms, Point of Zero Charge, and Isosteric Heat Studies. Adsorpt. Sci. Technol. 2023, 2023, 2813663 10.1155/2023/2813663.

[ref89] Li Z.; Mao Y.; Yan X.; Song Z.; et al. Design a Flower-like Magnetic Graphite Carbon Microsphere for Enhanced Adsorption of 2,4-Dichlorophenol. Environ. Sci. Pollut. Res. 2022, 29 (55), 83138–83154. 10.1007/s11356-022-21364-x. 35763142

[ref90] Liang J.; Liu J.; Yuan X.; Dong H.; Zeng G.; et al. Facile Synthesis of Alumina-Decorated Multi-Walled Carbon Nanotubes for Simultaneous Adsorption of Cadmium Ion and Trichloroethylene. Chem. Eng. J. 2015, 273, 101–110. 10.1016/j.cej.2015.03.069.

[ref91] Amran F.; Zaini M. A. A. Valorization of Casuarina Empty Fruit-Based Activated Carbons for Dyes Removal – Activators, Isotherm, Kinetics and Thermodynamics. Surfaces and Interfaces 2021, 25 (May), 101277 10.1016/j.surfin.2021.101277.

