# Supplementary material for: Casuarina equisetifolia-Derived Mesoporous Carbon for Adsorptive Removal of Phenolic Pollutants: Insights into Optimization, Isotherm, Kinetics, Thermodynamics, and Mechanism
Source: ACS Omega. 2025 Feb 28;10(9):8935–50. doi: 10.1021/acsomega.4c05796 (PMC11904702; doi:10.1021/acsomega.4c05796)
Supplement: Supplementary file 1 — ao4c05796_si_001.pdf [file ao4c05796_si_001.pdf]

Casuarina equisetifolia derived mesoporous carbon for adsorptive removal of phenolic pollutants: insights into optimisation, isotherm, kinetics, thermodynamics and mechanism

Praveengouda Patil[1], Jithu George Valiaparampil[1], Jaahnavi Urs[1], Gautham Jeppu[1*], Chikmagalur Raju Girish[1*]

Department of Chemical Engineering, Manipal Institute of Technology, Manipal Academy of Higher Education, Manipal

*Email id: cr.girish@manipal.edu

gautham.jeppu@manipal.edu

Supplementary File

1. Steps for calculation of adsorption isotherm parameters and constants in DR model

Table S1 Data pertaining to adsorption of 2,4-DCP on CEAC

C1	C2	C3	C4	C5	C6	C7	C8	C9	C10	C11
**Co (mg/L)**	**Ce (mg/L)**	**Qe**	**ln(qe)**	**R (J/mol/K)**	**T**	**1/Ce**	**1+1/Ce**	**ln(1+1/Ce)**	**ε**	**ε^2**
25	1.01101	23.988	3.17759			0.9891	1.9891	0.6876	1675.196	2806283
50	1.88705	48.112	3.87355			0.5299	1.5299	0.4252	1035.837	1072958
100	5.84573	94.154	4.54493	8.314	293	0.1710	1.1710	0.1579	384.6779	147977.1
150	10.3801	139.61	4.93892			0.0963	1.0963	0.0919	224.0517	50199.14
200	19.1019	180.89	5.19793			0.0523	1.0523	0.0510	124.3006	15450.63

The Table S1 lists all the experimental data and calculations. C1 to C11 represent column number. The values in C1 and C2 are initial and equilibrium concentration which are further used for compute adsorption capacity.. To obtain the parameters, initially

a) The graph of ln(qe) v/s ε^2 is plotted as shown in Fig. S1.

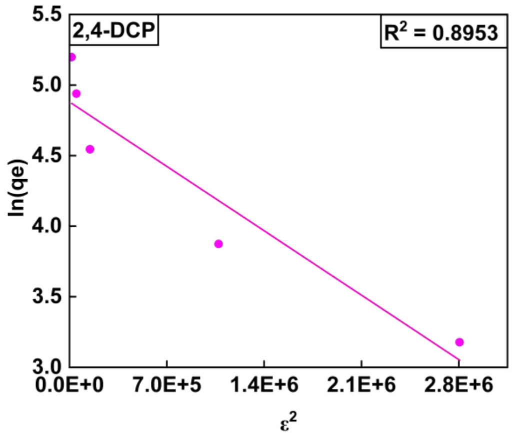

Fig. S1 Linear plot for DR model

b) Slope and intercept of the equation is either obtained using the inbuilt formulae of excel or linear trendline option.

c) The obtained equation is in the form of

y = -7E-07x + 4.8801
R^2 = 0.8953

Eq. 1

d) The equation 2 represents linear form of DR model.

$$Ln(q_e) = Ln(q_m) - k_{ad} * \varepsilon^2$$

Eq. 2

$$E = \frac{1}{\sqrt{2 * k_{ad}}}$$

e) Comparing equation 1 and 2 slope and intercept can be obtained.

Slope= -kd

Intercept= ln(qm)

f) Therefore,

	kd	7.00E-07	
ln(qm)=	intercept	-4.88	
	qm	0.007597	
	2kd	1.4E-06	
	Energy	845.15	kJ/mol

Similarly other isotherm parameters were calculated

2. Steps for calculation of adsorption kinetic parameters and constants

a) In case of kinetic experiments of 2,4-DCP adsorption, the investigation was performed for varied concentrations ranging from 25-200 mg/L till equilibrium was accomplished. The study involves drawing 2 ml of aliquots every 5 min till equilibrium contact time. The experimental values were further fitted to pseudo-first-order and pseudo-second-order kinetic models. The equations are represented as in Eq. 3 and Eq. 4

$$q_t = q_e(1 - e^{-k_1 t})$$

Eq. 3

$$q_t = \left(\frac{k_2 * q_e^2 * t}{1 + k_2 * q_e * t}\right)$$

Eq. 4

Table S2 Calculation to compute kinetic parameters in MS Excel

Adsorption Capacity (q_{exp})	Pseudo 1st order (PFO)	Pseudo 2nd Order (PSO)	Error functions					
			For (1st Order)			For (2nd Order)		
	q_t (mod)	q_t (mod)	SE	SR	APE	SE	SR	APE
17.722	16.142	17.342	2.498	27.076	0.089	0.144	27.076	0.021
19.787	21.133	20.151	1.812	9.850	0.068	0.133	9.850	0.018
20.836	22.677	21.301	3.387	4.364	0.088	0.216	4.364	0.022
21.765	23.154	21.927	1.928	1.346	0.064	0.026	1.346	0.007
22.361	23.302	22.321	0.885	0.319	0.042	0.002	0.319	0.002
22.751	23.347	22.591	0.355	0.030	0.026	0.026	0.030	0.007
23.077	23.361	22.788	0.081	0.023	0.012	0.083	0.023	0.013
23.158	23.366	22.938	0.043	0.054	0.009	0.048	0.054	0.009
23.004	23.367	23.056	0.132	0.006	0.016	0.003	0.006	0.002
23.158	23.367	23.152	0.044	0.054	0.009	0.000	0.054	0.000
23.385	23.368	23.442	0.000	0.211	0.001	0.003	0.211	0.002
23.554	23.368	23.591	0.035	0.395	0.008	0.001	0.395	0.002
23.779	23.368	23.681	0.169	0.729	0.017	0.010	0.729	0.004
23.722	23.368	23.741	0.126	0.634	0.015	0.000	0.634	0.001
23.895	23.368	23.817	0.279	0.941	0.022	0.006	0.941	0.003
23.880	23.368	23.862	0.262	0.911	0.021	0.000	0.911	0.001
23.846	23.368	23.893	0.229	0.848	0.020	0.002	0.848	0.002
23.830	23.368	23.915	0.214	0.819	0.019	0.007	0.819	0.004
23.783	23.368	23.931	0.173	0.736	0.017	0.022	0.736	0.006
24.012	23.368	23.944	0.415	1.180	0.027	0.005	1.180	0.003
23.919	23.368	23.954	0.304	0.988	0.023	0.001	0.988	0.001
23.982	23.368	23.963	0.378	1.117	0.026	0.000	1.117	0.001
24.077	23.368	23.970	0.503	1.326	0.029	0.012	1.326	0.004
Q_{avg} = 22.925		Sum	SSE=14.251	SSR=53.957	MAPE =0.0291	SSE=0.752	SSR= 53.957	MAPE = 0.006

$$R^2 = 1 - \frac{SSE}{SSR}$$

R^2= 0.73588 **R^2= 0.9861**

The experimental data was fitted to model by minimizing the errors (sum of square of errors) to obtain the rate constant k_1 = 1.174 1/min, q_e = 23.367 mg/g in case of PFO and k_1 = 0.108 g/mg/min, q_e = 24.047 mg/g in case of PSO of different concentrations (Table S2). Further, MAPE (mean absolute percentage error) was also evaluated.

From the Highest R^2 value and Lowest MAPE for PSO, it can be concluded that, PSO model better fits the adsorption data.

3. Steps for calculation of Thermodynamic parameters (ΔH°, ΔG°, ΔS°)

a) At the initial stage ln(Kd) is computed, Kd = Ca/Ce. The Table S3 incorporates detailed calculations for all the concentrations (25-200 mg/L) at varied temperatures (20 to 50 °C)

Where, Ca = Co-Ce

Table S3 Calculations to compute thermodynamic parameters

T °C	lnKd values for different concentrations at varied temperature (K)					
	1/T (K^{-1})	25 ppm	50 ppm	100 ppm	150 ppm	200 ppm
20	0.003413	3.1666	3.2385	2.7792	2.5990	2.2481
30	0.003300	2.6302	3.0522	2.7240	2.4314	1.9977
40	0.003195	2.3516	2.8612	2.6003	2.2186	1.8731
50	0.003096	2.1345	2.6382	2.1595	1.7173	1.4260

b) The graph of ln(K_D) v/s 1/T is plotted as shown in Fig. S2

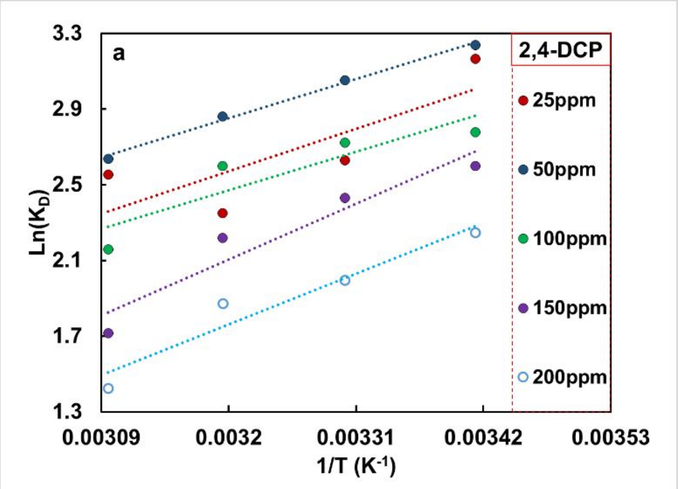

Fig. S2 Plot of ln(K_D) v/s 1/T for the calculations of thermodynamic parameters for the adsorption of 2,4-DCP

c) Slope and intercept of the equation is either obtained using the inbuilt formulae of excel or linear trendline option

d) The obtained equation pertaining to 50 mg/L adsorption is in the form of

$$y = 1881.8x - 3.1704$$

$$\Delta H = -R * Slope/1000$$

$$\Delta S = R * Intercept/1000$$

e) The obtained values of ΔH and ΔS were -15.64 kJ/mol and -0.026 kJ/mol respectively.

f) Similar calculation can be performed for other data as well

4. Steps for calculation of Thermodynamic parameters (ΔH_x)

a) Initially adsorption capacity for all Co and Ce values of varied concentrations are calculated.

b) The calculated values are tabulated in Table S4

c) The average of all the adsorption capacities is computed.

Table S4 Adsorption capacity of 2,4-DCP at different temperatures

Co	Adsorption capacity at different temperature				$Q_{exp,avg}$	$Q_{exp,avg}$ (mg/g) (rounded off)	Equilibrium concentrations at different temperature			
	Q_{exp} (20°)	Q_{exp} (30°)	Q_{exp} (40°)	Q_{exp} (40°)			C_e (20°)	C_e (30°)	C_e (40°)	C_e (40°)
25	23.989	23.319	22.826	22.355	23.123	23	1.01	1.68	2.17	2.64
50	48.112	47.744	47.295	46.664	47.453	47	1.89	2.26	2.71	3.34
100	94.154	93.843	93.088	89.656	92.685	90	5.85	6.16	6.91	10.34
150	139.62	137.879	135.287	127.168	134.988	130	10.38	12.12	14.71	22.83
200	180.89	176.11	173.364	161.256	172.907	170	19.10	23.89	26.64	38.74

d) Further a graph of ln(Ce) v/s 1/T for varied loading (mg/g) is plotted as shown in the Fig. S3

e) From the MS Excel, a set of linear trendlines are obtained for the points plotted.

f) A linear trendline is obtained from the plot with pertaining to 23 mg/g loading

$$y = -2991x + 10.294$$

g) From the above line, slope is used to compute ΔH_x

$$\Delta H_x = -Slope*R/1000$$

Where R = 8.314 J/K/mol

h) The obtained values of ΔH_x = 24.86 kJ/mol < 80 kJ/mol indicating physical adsorption dominating the removal process.

i) Similar calculations were also performed for other loadings.

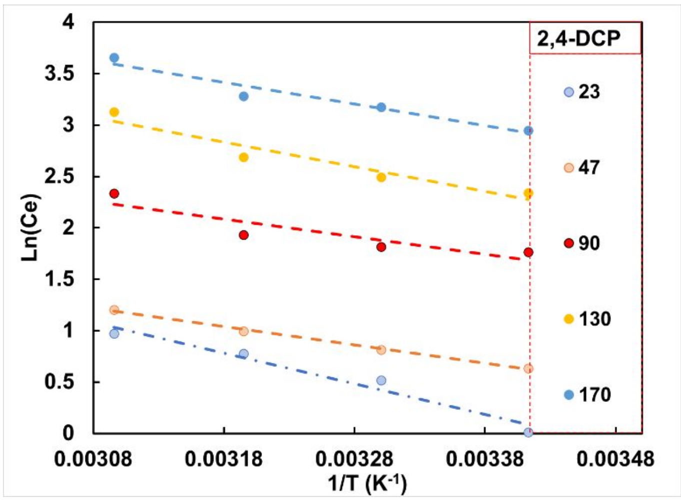

Fig. S3 plot of ln(Ce) v/s 1/T at varied loading for estimating ΔH_X
